# The Jasmine (*Jasminum sambac*) Genome Provides Insight into the Biosynthesis of Flower Fragrances and Jasmonates

**DOI:** 10.1016/j.gpb.2022.12.005

**Published:** 2022-12-29

**Authors:** Gang Chen, Salma Mostafa, Zhaogeng Lu, Ran Du, Jiawen Cui, Yun Wang, Qinggang Liao, Jinkai Lu, Xinyu Mao, Bang Chang, Quan Gan, Li Wang, Zhichao Jia, Xiulian Yang, Yingfang Zhu, Jianbin Yan, Biao Jin

**Affiliations:** 1 College of Horticulture and Plant Protection, Yangzhou University, Yangzhou 225009, China; 2 College of Bioscience and Biotechnology, Yangzhou University, Yangzhou 225009, China; 3 Department of Floriculture, Faculty of Agriculture, Alexandria University, Alexandria 21526, Egypt; 4 Key Laboratory of Plant Functional Genomics of the Ministry of Education, Yangzhou University, Yangzhou 225009, China; 5 Shenzhen Branch, Guangdong Laboratory for Lingnan Modern Agriculture, Key Laboratory of Synthetic Biology, Ministry of Agriculture and Rural Affairs, Agricultural Genomics Institute at Shenzhen, Chinese Academy of Agricultural Sciences, Shenzhen 518120, China; 6 College of Landscape Architecture, Nanjing Forestry University, Nanjing 210037, China; 7 Institute of Plant Stress Biology, State Key Laboratory of Cotton Biology, Department of Biology, Henan University, Kaifeng 475001, China

**Keywords:** Jasmine flower, *Jasminum sambac*, Genome, Jasmonate, Flower fragrance

## Abstract

***Jasminum sambac*** (**jasmine flower**), a world-renowned plant appreciated for its exceptional **flower fragrance**, is of cultural and economic importance. However, the genetic basis of its fragrance is largely unknown. Here, we present the first *de novo***genome** assembly of *J. sambac* with 550.12 Mb (scaffold N50 = 40.10 Mb) assembled into 13 pseudochromosomes. Terpene synthase (TPS) genes associated with flower fragrance are considerably amplified in the form of gene clusters through tandem duplications in the genome. Gene clusters within the salicylic acid/benzoic acid/theobromine (SABATH) and benzylalcohol *O*-acetyltransferase/anthocyanin *O*-hydroxycinnamoyltransferases/anthranilate *N*-hydroxycinnamoyl/benzoyltransferase/deacetylvindoline 4-*O*-acetyltransferase (BAHD) superfamilies were identified to be related to the biosynthesis of phenylpropanoid/benzenoid compounds. Several key genes involved in **jasmonate** biosynthesis were duplicated, causing an increase in copy numbers. In addition, multi-omics analyses identified various aromatic compounds and many genes involved in fragrance biosynthesis pathways. Furthermore, the roles of *JsTPS3* in β-ocimene biosynthesis, as well as *JsAOC1* and *JsAOS* in jasmonic acid biosynthesis, were functionally validated. The genome assembled in this study for *J. sambac* offers a basic genetic resource for studying floral scent and jasmonate biosynthesis, and provides a foundation for functional genomic research and variety improvements in *Jasminum*.

## Introduction

*Jasminum sambac* (common names: Arabian jasmine, Sambac jasmine, jasmine flower, and Mo-Li-Hua) is famous worldwide as a fragrant plant with sweet-scented flowers. The fragrant flowers of *J. sambac* are used for the extraction of jasmine essential oil, which is a common natural ingredient in the perfume and cosmetic industries, as well as in pharmaceutical applications and aromatherapy [1], [2]. *J. sambac* flowers are also used in the manufacture of jasmine tea, which is widely consumed in East Asia [3], [4]. Various food products with the sweet flavor of *J. sambac* flowers have been produced, such as syrup, aerated water, jam, yogurt, ice cream, and wine. In some Asian countries, *J. sambac* is regarded as an auspicious symbol in religious ceremonies or used to make garlands for welcoming guests [5], and it has been integrated into local cultures and traditions [6], [7].

Flower fragrances are volatile organic compounds (VOCs) emitted by flowering plants to attract pollinators and ensure reproductive success [8]. Flower fragrances also attract humans and have become the focus of intensive use in the floriculture and fragrance industries. Different flowering plant species have distinct flower fragrances, depending on differences in the composition, amount, and emission of floral VOCs [8], [9]. The VOCs of *J. sambac* floral scents belong mainly to the terpenoid and phenylpropanoid/benzenoid classes [10]. However, most previous analyses of VOCs from *J. sambac* flowers were based on harvested flowers [11], [12], [13]; the fragrances actively released by flowers growing in the natural state remain obscure. Some genes involved in the biosynthesis pathways of *J. sambac* floral scent compounds have been isolated and analyzed, including genes responsible for the biosynthesis of α-farnesene (*JsHMGS*, *JsHMGR*, *JsFPPS*, and *JsTPS*) in the mevalonic acid (MVA) pathway [14]. However, the biosynthesis pathways of floral scent compounds and their regulatory networks are complex, and their underlying genetic mechanisms remain largely unknown. Whole-genome sequencing in combination with transcriptomics and metabolomics analyses is a practical strategy for identifying the metabolic pathways of natural compound biosynthesis in plants [15], [16], [17]. Although *J. sambac* flower products are widely used and their flower scents are economically valuable, the lack of *J. sambac* genome data seriously hampers progress in unraveling the biosynthesis and metabolism of its fragrance. In addition, jasmonates are important aromatic substances in *Jasminum* flowers. Jasmonates have been extensively studied in the context of biotic and abiotic stress responses and defenses in model plants, crops, and other plants [18]. However, research on jasmonate biosynthesis and regulation in *Jasminum* is impeded by the absence of genome sequence data.

Here, we reported a chromosome-level genome assembly of *J. sambac* obtained using a combination of PacBio and Hi-C sequencing technologies. Furthermore, by combining multi-omics analyses of different stages of flowers, we identified various aromatic compounds released from both harvested and naturally grown flowers. Several important genes involved in the biosynthesis pathways of major fragrant compounds in jasmine flowers were identified and validated. This *J. sambac* genome sequence and the identified floral scent volatiles offer valuable resources for *J. sambac* genetic research, and will lay a foundation for biological and agronomic research on this commercially and culturally important species.

## Results

**Figure 1:**
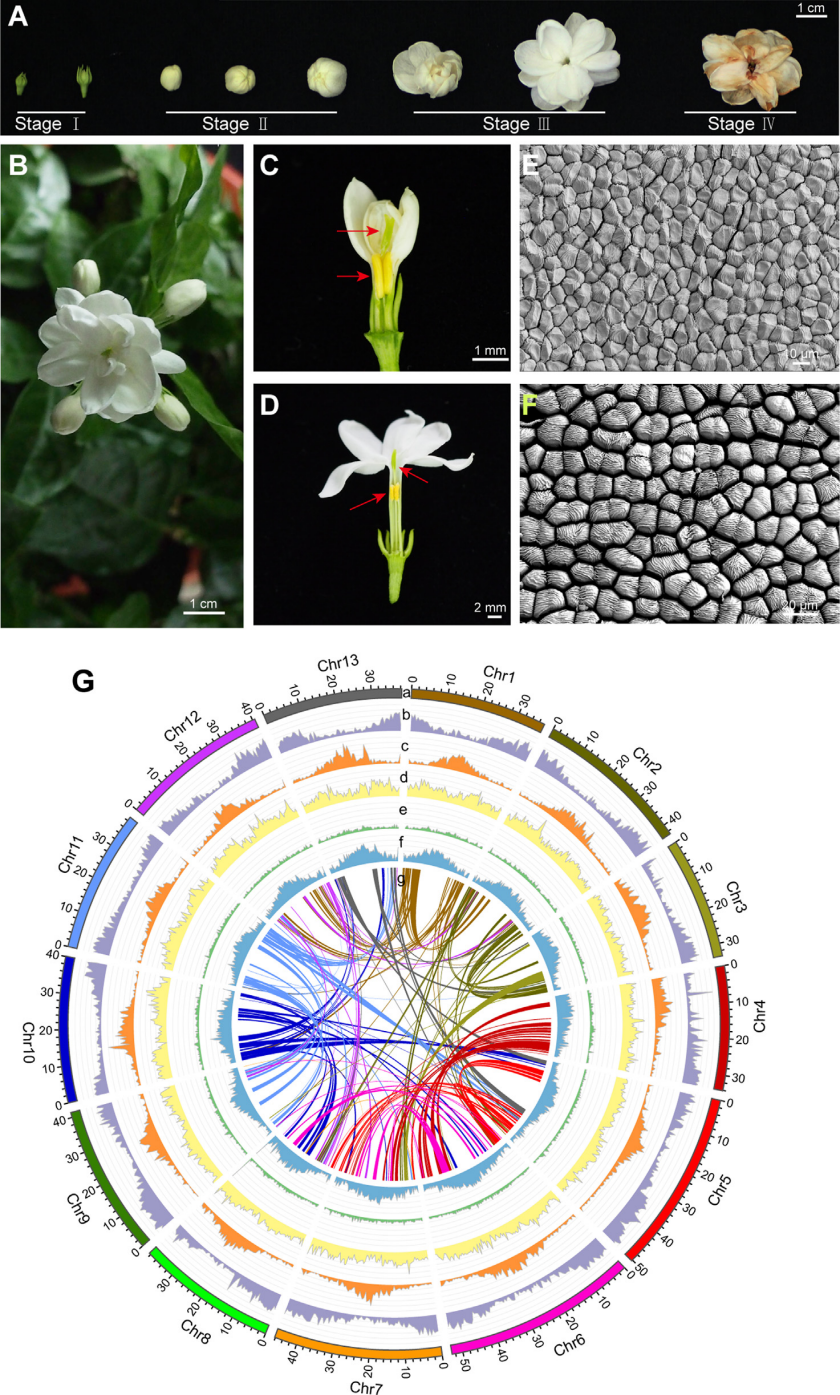
**Morphology of flowers and genomic features of *J. sambac*** **A.** Different stages of *J. sambac* flowers. **B.** Morphology of the FF. **C.** Anatomy of the FB. **D.** Anatomy of the FF. Red arrows indicate the pistil and stamens. **E.** Morphology of the petal cells of a FB under scanning electron microscope. **F.** Morphology of the petal cells of a FF under scanning electron microscope. **G.** Genomic features. a, circular representation of the 13 pseudochromosomes (Mb); b, gene density; c, *Gypsy* density; d, *Copia* density; e, DNA TE density; f, total TE density; g, syntenic relationships among duplication blocks containing more than 13 paralogous gene pairs. The y-axis of b−f in (G) indicates the gene density or TE density in a sliding window (bin size = 500 kb). TE, transposable element; FF, full-bloom flower; FB, flower bud.

In this study, we used the cultivar ‘double petal’ (the most widely cultivated *J. sambac*) for genome sequencing (Figure 1A and B). We also used the leaves, flower buds (FBs, stage II; Figure 1C and E), and full-bloom flowers (FFs, including semi-bloom to full-bloom, stage III; Figure 1D and F) for transcriptomic and metabolomic analyses, to identify various aromatic compounds and key genes involved in fragrance biosynthesis pathways.

### A chromosome-level genome assembly of *J. sambac*

**Supplementary Figure S1:**
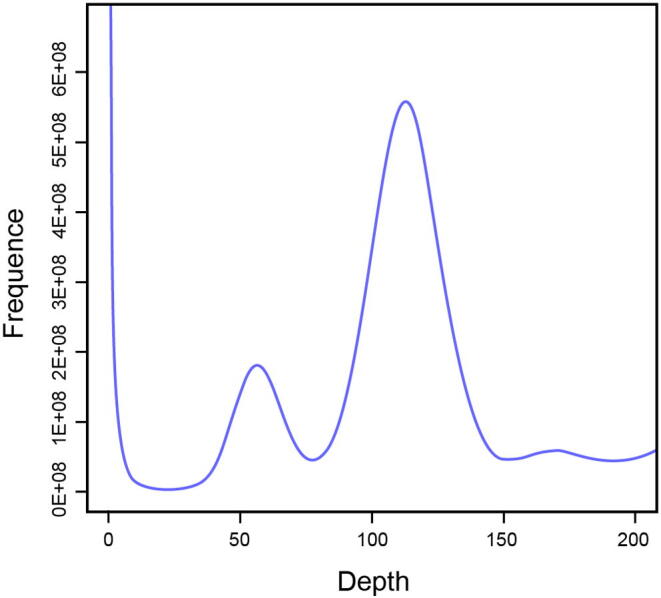
**Evaluation of the genome size of *J. sambac* by 17-mer analyses** The distribution of 17-mer depth of high-quality reads. The x-axis represents the sequencing depth and the y-axis represents the frequency of the 17-mers at a given sequencing depth.

*k*-mer analysis of the second-generation short reads (89.05 Gb used) revealed that the *J. sambac* genome size is ∼ 573.02 Mb with a heterozygosity rate of 0.99% and a repeat proportion of 57.12% (Figure S1). The genome was sequenced and assembled using a combination of single-molecule real-time (SMRT) sequencing technology from PacBio and Hi-C. In total, 63.82 Gb of PacBio long reads (∼ 116× coverage of the assembled genome) were generated from 4.5 million PacBio single-molecule long reads (average read length = 14.2 kb, longest read length = 127.0 kb). PacBio long reads were assembled using the overlap-layout-consensus (OLC) method. After multiple Quiver corrections (PacBio long reads), Pilon corrections (Illumina short reads for *k*-mer analysis), and haplotig purging, *de novo* assembly yielded 373 contigs with a contig N50 length of 2.50 Mb. The total assembly size was 550.12 Mb, with a GC content of 34.62% and covering 96% of the estimated *J. sambac* genome size (Table S1).

To refine the *J. sambac* assembly, Hi-C libraries were constructed and sequenced using DNA from the leaves of *J. sambac*. In total, we generated 61.6 Gb of Illumina paired-end reads (∼ 112× coverage of the assembled genome). The Hi-C read pairs were mapped onto the draft assembly and used to improve the scaffold N50 length to 40.10 Mb and the contig number to 383, with the longest scaffold being 52.97 Mb and the scaffold number being 112 (Tables S1 and S2). The final reference assembly consisted of 13 chromosome-scale pseudomolecules (the pseudomolecules are hereafter referred to as chromosomes) (Figure 1G), with maximum and minimum lengths of 52.97 Mb and 33.49 Mb, respectively (Table S3). The total length of the chromosomes accounts for 97.36% (535.57 Mb) of the assembled genome size of 550.12 Mb.

**Supplementary Figure S2:**
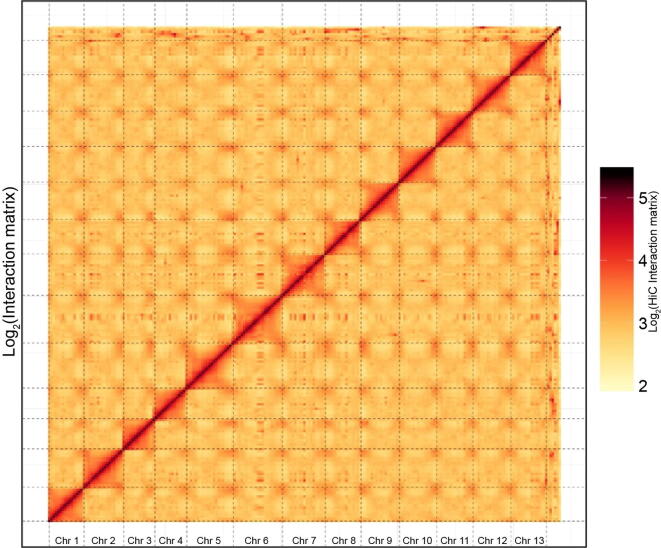
**Hi-C interaction heatmap of the *J. sambac* reference genome showing interactions among the 13 chromosomes** Hi-C reads were realigned back to the *J. sambac* assembled genome. The intensity of pixels represents the count of Hi-C links, which indicates the likelihood of loci collocating in the nucleus. Darker red color indicates higher contact probability. Bin size, 250 Kb.

To evaluate the quality of the assembled genome, benchmarking universal single-copy orthologs (BUSCO) [19] assessment was conducted, and the results revealed a high-quality draft genome covering 1569 (97.20%; Table S4) complete orthologs of the 1614 plant-specific sequences (embryophyta dataset from BUSCO). Long terminal repeat (LTR) assembly index (LAI) evaluation showed that the value of LAI is 23.53% for *J. sambac* genome, suggesting a more complete assembly achieving gold quality (LAI ≥ 20) [20]. Core eukaryotic genes mapping approach (CEGMA) evaluation showed that 93.95% (233 of 248 conserved genes in core gene library constructed by six eukaryotic model organisms) core eukaryotic genes (CEGs) was obtained. Furthermore, the mapping rates of the DNA-seq and RNA-seq datasets ranged from 86.25% to 93.40%. In addition, a Hi-C interaction heatmap separated distinct regions on 13 different chromosomes (Figure S2). These results demonstrate that the assembled *J. sambac* genome is of high quality at the chromosome level.

### Genome annotation reveals the repetitive sequence landscape

**Supplementary Figure S3:**
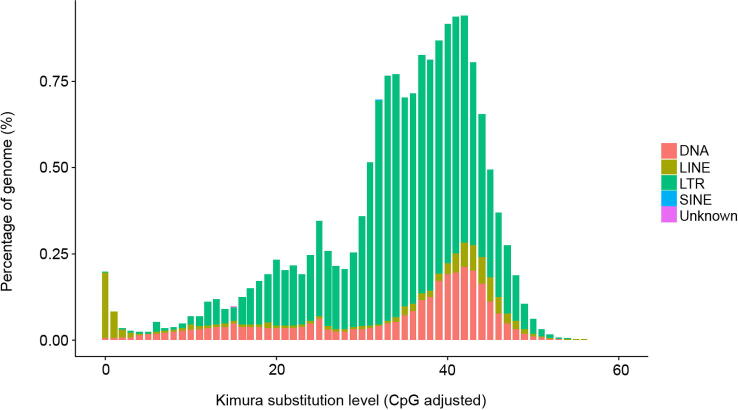
**Divergence distribution of transposable elements in the genome of *J. sambac*** Data represent the Kimura substitution level (CpG-adjusted). LINE, long interspersed nuclear elements; SINE, short interspersed nuclear elements; LTR, long terminal repeat.

We identified a total of 259.8 Mb of repetitive sequences in the genome of *J. sambac*, which accounted for 47.68% of the assembled genome. Among them, LTR retrotransposons were the predominant transposable element (TE) components accounting for 35.06% of the assembled genome, followed by DNA TEs (6.8%) (Table S5). Within the LTR order, the *Copia* superfamily was the most abundant, accounting for 16.9% of the genome, followed by the *Gypsy* superfamily (15.04%), consequently showing a low *Gypsy*/*Copia* ratio (0.89) in *J. sambac*. In addition, the distribution of TEs varied across the genome (Figure 1G, Figure S3). For example, the TE content was higher near the centromeres compared to other parts of the chromosomes.

**Supplementary Figure S4:**
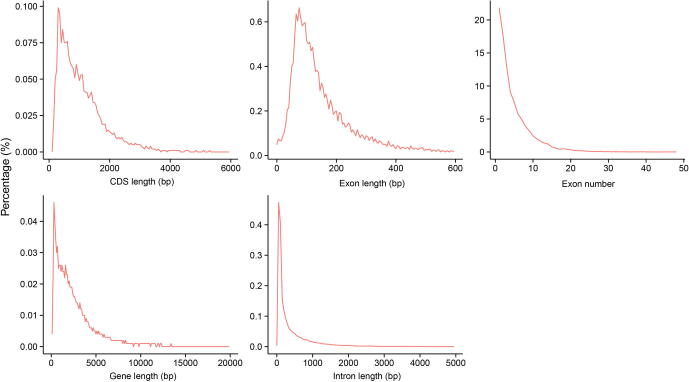
**Different elements of annotated genes in the *J. sambac* genome** The distributions of CDS length, exon length, exon number per gene, gene length, and intron length are shown. CDS, coding sequence.

**Supplementary Figure S5:**
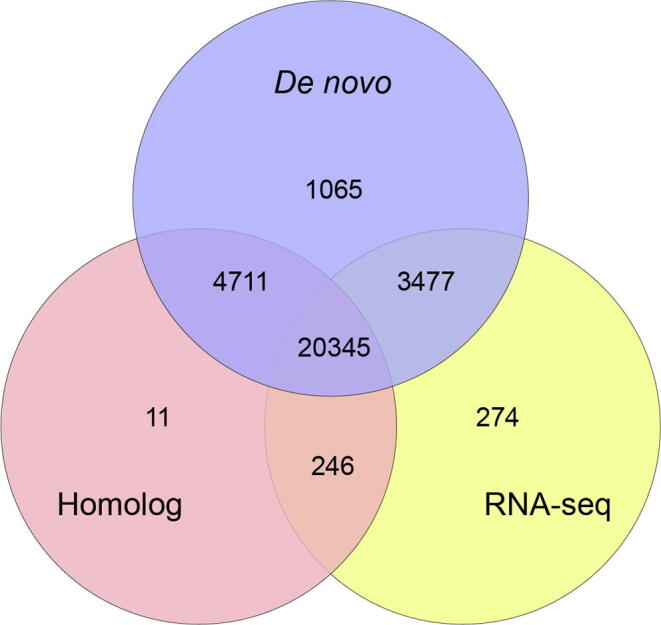
**Summary of protein-coding genes in the *J. sambac* genome predicted by *de novo*, homology-based, and RNA-Seq approaches** All gene models predicted from the above three approaches were combined by EVM into a non-redundant set of gene structures. The low-quality gene models were filtered based on the criterion of genes overlapping by more than 50%. EVM, EVidenceModeler.

We annotated the remaining repeat-masked *J. sambac* genome using a comprehensive strategy of *de novo* prediction combined with homology-based and transcriptome-based protein predictions. In total, 30,129 complete genes were predicted, with an average transcript length of 3336 bp and an average coding sequence (CDS) length of 1104 bp (Figure S4; Tables S1, S6, and S7). Among the predicted genes, 67.5% (20,345/30,129) were predicted by all three strategies (Figure S5). BUSCO analysis showed that relatively high completeness of genome annotation covering 1462 (90.60%; Table S8) complete orthologs of the 1614 plant-specific sequences.

**Supplementary Figure S6:**
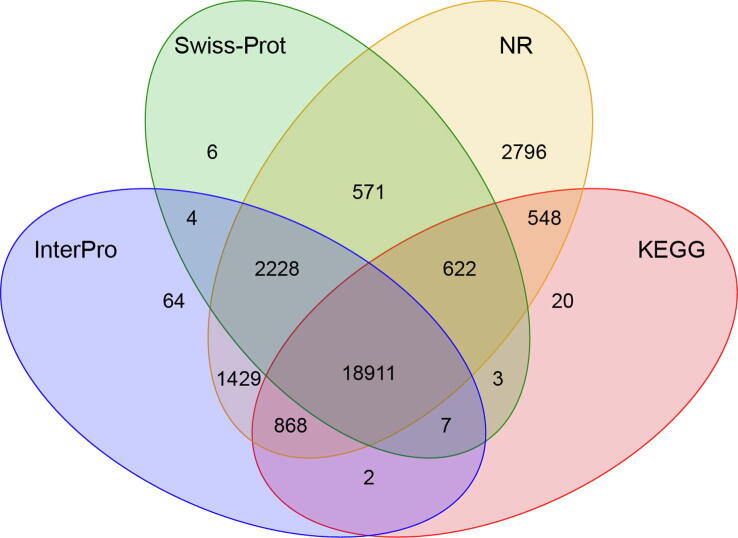
**Functional annotation of genes in the *J. sambac* genome** The protein sequences predicted by gene structure analysis were identified in the Swiss-Prot, InterPro, NR, and KEGG databases. NR, Non-Redundant Protein Sequence Database; KEGG, Kyoto Encyclopedia of Genes and Genomes.

In total, 9902 non-coding RNAs, including 1657 microRNAs (miRNAs), 1767 ribosomal RNAs (rRNAs), and 535 transfer RNAs (tRNAs), were identified (Table S9). Further functional annotation revealed that 93.20% of all predicted genes could be annotated with the following protein-related databases: non-redundant (NR) database (92.80%), Swiss-Prot (74.20%), Kyoto Encyclopedia of Genes and Genomes (KEGG) (69.60%), InterPro (78.00%), Gene Ontology (GO) (53.80%), and Pfam (73.00%) (Table S10). In total, 18,911 genes were commonly annotated in the Swiss-Prot, InterPro, NR, and KEGG databases (Figure S6).

### Phylogenetic positioning and genome evolution of *J. sambac*

**Figure 2:**
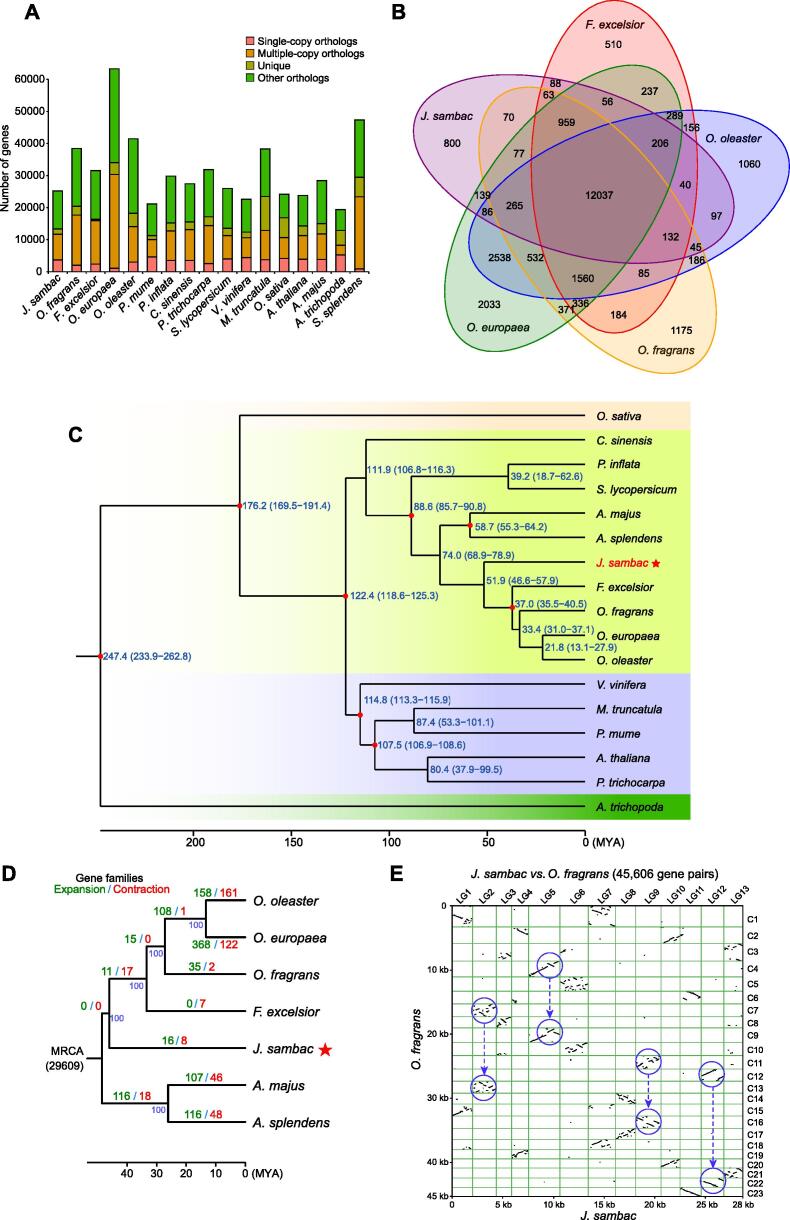
**Comparative genomic analysis reveals phylogenetic positioning and genome evolution of *J. sambac*** **A.** Distribution of genes in *J. sambac* and 16 representative plant species. Only the longest isoform for each gene was used. Gene clusters (families) were identified using the OrthoMCL package with the default parameters. **B.** Venn diagram showing the shared orthologous gene families in *J. sambac*, *F. excelsior*, *O. fragrans*, *O. europaea*, and *O. oleaster.* The number of gene families is listed for each component. **C.** Phylogenetic tree constructed from single-copy gene families of *J. sambac* and 16 representative plant species. The red dot on the node indicates the calibration points. The blue numbers beside each node indicate the divergence time of each species. **D.** Expansion and contraction of gene families. The numerical value beside each node shows the numbers of expanded (green) and contracted (red) gene families. The blue numbers under the branches represent bootstrap values. For phylogenetic analyses (C and D), multiple sequence alignment of single-copy orthologous genes was performed by MUSCLE, and the phylogenetic tree was constructed using RAxML. **E.** Syntenic dot plot between *J. sambac* and *O. fragrans* using 45,606 homologous gene pairs. The blue circles indicate no additional WGD event (one *vs.* two collinearity fragments) occurring in *J. sambac* after the divergence of *J. sambac* and *O. fragrans*. WGD, whole-genome duplication; MRCA, most recent common ancestor; *O. fragrans*, *Osmanthus fragrans*; *F. excelsior*, *Fraxinus excelsior*; *O. europaea*, *Olea europaea*; *O. oleaster*, *Olea oleaster*.

**Supplementary Figure S7:**
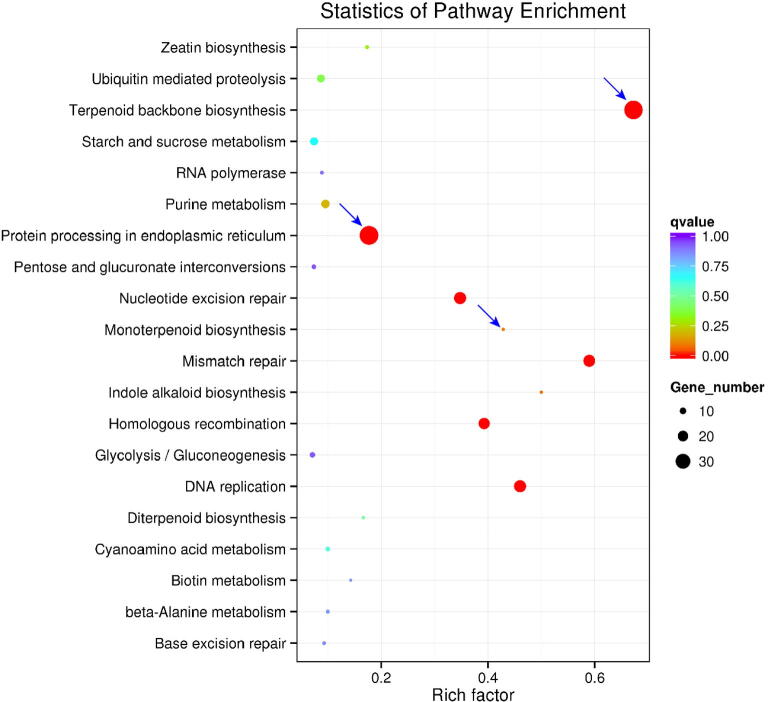
**KEGG enrichment analysis of the *J. sambac*-specific gene families** Blue arrows show the enriched items important for volatile compound biosynthesis in *J. sambac* flower.

**Supplementary Figure S8:**
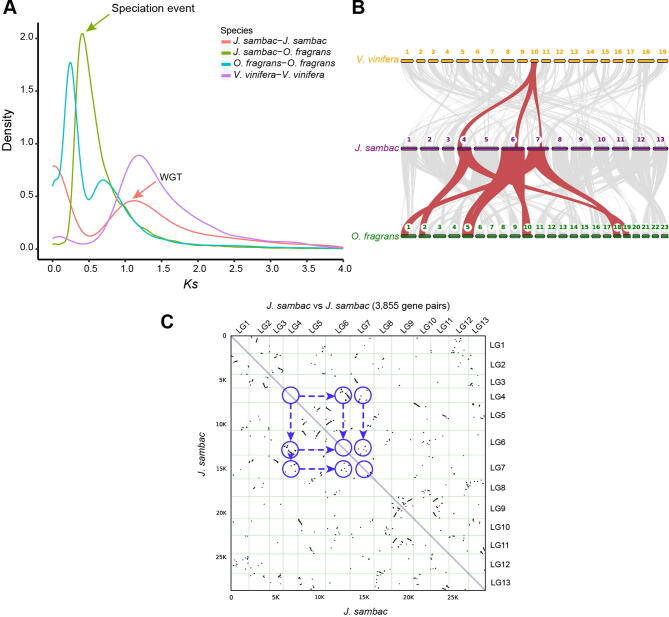
**WGT event in Oleaceae A.***Ks* density profiles of *J. sambac*, *O. fragrans*, *V. vinifera*, and *J. sambac vs. O. fragrans*. **B.** Synteny block among the genomes of *V. vinifera*, *J. sambac*, and *O. fragrans.***C.** Dot plot of synteny within *J. sambac* genome. *Ks*, synonymous substitution rate.

The evolutionary dynamics of gene families were analyzed by comparing the *J. sambac* genome with those of 16 representative plant species. In total, 42,577 gene families were clustered among all 17 species, and 6337 gene families were in common, including 3670 single-copy orthologs (Figure 2A). From the gene families clustered in five species of the Oleaceae family (*J. sambac*, *Osmanthus fragrans*, *Fraxinus excelsior*, *Olea europaea*, and *Olea oleaster*), 15,160 gene families were identified in the *J. sambac* genome, of which 800 gene families (2060 genes) were *J. sambac*-specific whereas 12,037 gene families were shared among all five species within the family (Figure 2B). Functional enrichment analysis of the *J. sambac*-specific gene families indicated that these gene families are mainly involved in terpenoid backbone biosynthesis, monoterpenoid biosynthesis, and protein processing in the endoplasmic reticulum (Figure S7), which are likely important for volatile compound biosynthesis in *J. sambac* flowers [10]. A phylogenetic tree was constructed from single-copy gene families of *J. sambac* and the 16 representative plant species (Figure 2C), revealing that the families Oleaceae and Labiatae split ∼ 74.0 million years ago (MYA), whereas *J. sambac* diverged ∼ 51.9 MYA from other Oleaceae members (*i.e.*, earlier than the other four species). In addition, we found 16 expanded gene families and 8 contracted gene families considerably in *J. sambac* compared to the common ancestor of Oleaceae and Labiatae (Figure 2D). The expanded gene families were involved mainly in riboflavin metabolism and butanoate metabolism (both related to fragrant volatiles). One contracted gene family was related to plant–pathogen interactions (Table S11). We applied synonymous substitution rate (*Ks*) and syntenic analyses to detect whole-genome duplication (WGD) events (Figure S8A). The results showed a notable peak (1.1–1.2 MYA and 46.2–55.0 MYA) in the *J. sambac Ks* profile, which was larger than *O. fragrans* but smaller than *Vitis vinifera*, indicating an ancient WGD event existing in the *J. sambac* genome. We also compared the *Ks* profiles of *J. sambac* and *O. fragrans* and concluded that the ancient WGD event occurred in the common ancestor before their divergence (Figure S8A).

We further conducted muti-species collinearity analysis among *V. vinifera*, *J. sambac*, and *O. fragrans* genomes (Figure S8B). Because it is widely believed that *V. vinifera* genome has experienced ancestral whole-genome triplication (WGT)-γ event [21], three *vs.* one collinearity fragments between the *J. sambac* and *V. vinifera* genomes (Figure S8B) indicated the existence of an additional WGT event in *J. sambac* genome after the ancestral WGT-γ. Furthermore, a dot plot of syntenic analysis (Figure S8C) showed that the *J. sambac* genome had experienced a WGT event. In addition, by comparing the genomes of *J. sambac* and *O. fragrans* using 45,606 homologous gene pairs, we did not find any additional WGD event (one *vs.* two collinearity fragments) in *J. sambac* after the divergence between *J. sambac* and *O. fragrans* (Figure 2E). This finding is consistent with the aforementioned results revealed by *Ks* profiles.

### Expansion of the terpene synthase gene family contributes to terpene biosynthesis

**Figure 3:**
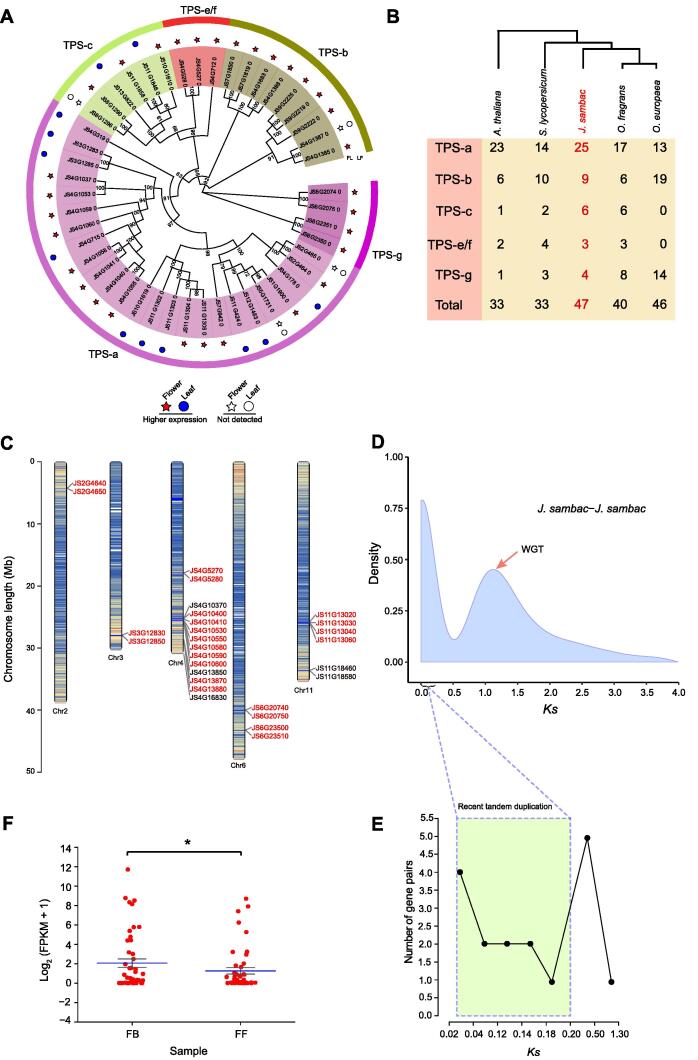
**Expansion and clusteringof *TPS* genes in *J. sambac* genome** **A.** Phylogenetic tree of the 47 *TPS* genes containing two conserved domains identified in the *J. sambac* genome. Colored stars and circles indicate the *TPS* genes highly expressed in flowers (FBs and FFs) and leaves, respectively. The expression level of each *TPS* gene is presented in Table S12. TPS protein sequences were aligned using Clustal X (version 2.0), and the phylogenetic tree was constructed using IQ-TREE (version 1.6.9) by the maximum likelihood method with bootstrap values of 1000 replicates. The black numbers beside the branches represent bootstrap values. **B.** Number of *TPS* genes containing two conserved domains in *J. sambac*, *A. thaliana*, *S. lycopersicum*, *O. fragrans*, and *O. europaea*. **C.** Chromosomal distribution of the *J. sambac TPS* genes. The colored lines in different chromosomes indicate the gene density; darker lines indicate higher gene density. Red gene IDs indicate *TPS* genes that form gene clusters on chromosomes. The chromosomal distribution of *TPS* genes was illustrated with TBtools. **D.***Ks* distribution of the paralogous *TPS* genes in the *J. sambac* genome. **E.** The *TPS* gene pairs (paralogs) in the box had *Ks* < 0.2. **F.** Expression levels of *TPS* genes in FBs and FFs of *J. sambac.* Blue bars indicate the mean expression levels. *, *P* < 0.05 (Student’s *t*-test). TPS, terpene synthase; FPKM, fragments per kilobase million; *Ks*, synonymous substitution rate.

**Figure 4:**
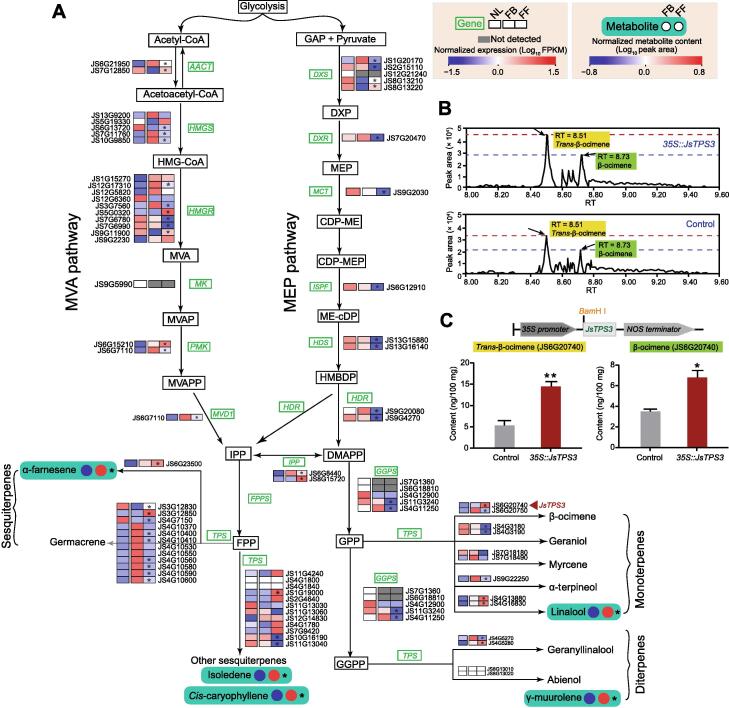
**Genes and metabolites involved in terpenebiosynthesis pathways in *J. sambac*** **A.** Heatmaps showing the expression levels of genes (in green) involved in terpene biosynthesis pathways. Circles to the right of metabolites (highlighted in the cyan backgrounds) indicate the different contents of metabolites in FBs (left) and FFs (right). Asterisks (*) indicate significant differences in gene expression or metabolite content between FBs and FFs. DEGs were identified based on adjusted *P* < 0.05 and |log_2_ fold change| > 1. Differentially abundant metabolites were determined based on *P* < 0.05 and VIP ≥ 1 for UPLC–ESI–MS/MS and GC–MS, or FDR < 0.05 for volatile metabolites detected from flowers on living plants by GC–MS. The red triangular arrowhead indicates that the *JsTPS3* gene (JS6G20740) was significantly up-regulated in FFs. The expression levels of genes and the contents of metabolites were normalized, with red and blue indicating high and low gene expression/metabolite content, respectively. FBs, FFs, and NLs were sampled from three biological replicates. **B.** GC–MS peaks of *trans*-β-ocimene and β-ocimene in *JsTPS3*-transgenic *J. sambac* calli. **C.***Trans*-β-ocimene and β-ocimene contents in *JsTPS3*-transgenic *J. sambac* calli. Data are shown as mean ± SD from five biological replicates. *, *P* < 0.05; **, *P* < 0.01 (Student’s *t-*test). NL, normal leaf; SD, standard deviation; acetyl-CoA, acetyl coenzyme A; acetoacetyl-CoA, acetoacetyl coenzyme A; HMG-CoA, 3-hydroxy-3-methylglutaryl coenzyme A; MVA, mevalonic acid; MVAP, mevalonic acid-5-phosphate; MVAPP, mevalonic acid-5-diphosphate; GAP, glyceraldehyde-3-phosphate; DXP, 1-deoxy-D-xylulose 5-phosphate; MEP, 2-*C*-methylerythritol 4-phosphate; CDP-ME, 4-diphosphocytidyl-2-*C*-methyl-D-erythritol; ME-cDP, 2-*C*-methyl-D-erythritol-2,4-cyclodiphosphate; HMBDP, 1-hydroxy-2-methyl-2-(E)-butenyl 4-diphosphate; IPP, isopentenyl pyrophosphate; DMAPP, dimethylallyl diphosphate; FPP, farnesyl diphosphate; GPP, geranyl pyrophosphate; GGPP, geranylgeranyl diphosphate; AACT, acetoacetyl-CoA thiolase; HMGS, HMG-CoA synthase; HMGR, HMG-CoA reductase; MK, mevalonic acid kinase; PMK, phosphomevalonate kinase; MVD1, mevalonate diphosphate decarboxylase; DXS, 1-DXP synthase; DXR, DXP reducto-isomerase; MCT, 2-*C*-methyl-D-erythritol-4-(cytidine-5-diphospho) transferase; ISPF, ME-cDP synthase; HDS, HMBDP synthase; HDR, HMBDP reductase; FPPS, FPP synthase; GGPS, GGPP synthase; DEG, differentially expressed gene; RT, retention time; VIP, variable importance in projection; FDR, false discovery rate; UPLC–ESI–MS/MS, ultra-performance liquid chromatography–electrospray ionization–tandem mass spectrometry; GC–MS, gas chromatography–mass spectrometry.

**Supplementary Figure S9:**
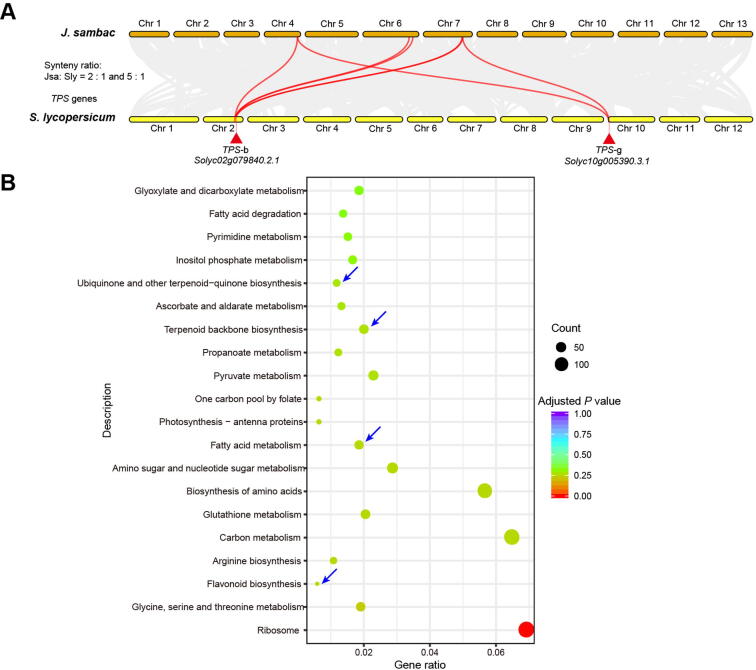
**Synteny and KEGG enrichment analyses A.** Synteny analysis of the genomes of *J. sambac* and *S. lycopersicum*. The TPS-b and TPS-g subfamily genes in *S. lycopersicum* showed 1 (*Solyc10g005390.3.1*) : 2 (*JS4G13880, JS7G18500*) and 1 (*Solyc02g079840.2.1*) : 5 (*JS4G13850, JS6G23500, JS6G20740, JS7G18500,* and *JS7G18190*) collinear relationships (red lines) to *J. sambac* TPS genes at the chromosome level. Gray lines in the background indicate the collinear blocks within *J. sambac* and *S. lycopersicum* genomes. B. KEGG enrichment analysis of DEGs in FBsand FFs. The most important KEGG pathways are denoted by the blue arrows. DEGs, differentially expressed genes. TPS, terpene synthase; DEG, differentially expressed genes; FB, flower buds; FF, full-bloom flowers.

**Supplementary Figure S10:**
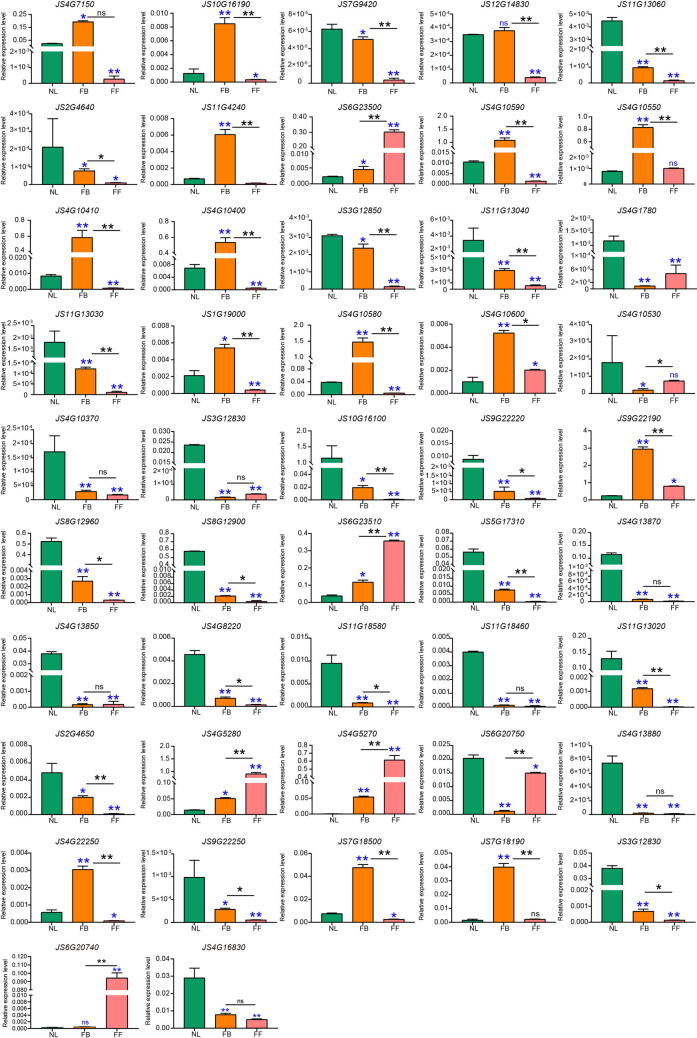
**qRT-PCR results for all 47 TPSs in *J. sambac*** qRT-PCR results of all TPS genes (with two conserved domains) in NL, FBs, and FFs in *J. sambac*. Blue asterisks indicate significant differences relative to NL: *, *P* < 0.05, **, *P* < 0.01 (one-way ANOVA with Dunnett’s post hoc test). Black asterisks indicate significant differences between FBs and FFs: *, *P* < 0.05, **, *P* < 0.01 (one-way ANOVA with Dunnett’s post hoc test). qRT-PCR, quantitative reverse-transcription polymerase chain reaction; NL, normal leaves.

The terpene synthase (TPS) family is a vital enzyme gene family for terpene biosynthesis, which is crucial in the production of floral VOCs. We identified 59 candidate *TPS* genes in *J. sambac* containing at least one conserved domain, and most of the *TPS* genes (47 of 59) contained two conserved domains (Table S12). We further constructed an evolutionary tree of the 47 *TPS* genes containing two conserved domains and found that the *TPS* genes of *J. sambac* could be classified into five subgroups: TPS-a, TPS-b, TPS-c, TPS-e/f, and TPS-g. TPS-a was the largest subgroup, accounting for 53.2% of the total *TPS* genes (Figure 3A). The number of *TPS* genes containing two conserved domains was considerably higher in *J. sambac* than in *Arabidopsis thaliana* (33), *Camellia sinensis* (30), *Solanum lycopersicum* (33), *O. fragrans* (40) (Figure 3B), cacao (*Theobroma cacao*; 36), and kiwifruit (*Actinidia chinensis*; 34). Almost half of the *J. sambac TPS* genes (23 of 47) were derived from tandem duplication, and these genes formed *TPS* gene clusters on chromosomes 2, 3, 4, 6, and 11, which were commonly annotated as (-)-germacrene D synthase, geranyl linalool synthase, and (E)-beta-ocimene synthase (red gene IDs in Figure 3C). Through phylogenic analysis of the *TPS* genes, we further identified 17 gene pairs (paralogs), 11 of which had *Ks* < 0.2 (Figure 3D and E), smaller than the *Ks* value of WGD peak. This result implies that the *TPS* gene duplications are relatively recent events (rather than resulting from a WGD event) that occurred after the speciation of *J. sambac*, contributing to the amplification of *TPS* genes in the *J. sambac* genome. Notably, we also found several events of 5:1 or 2:1 replication of *TPS* genes between the *J. sambac* and tomato genomes (Figure S9A), indicating the expansion of the TPS family in *J. sambac* (Oleaceae) relative to tomato (Solanaceae). Furthermore, the differentially expressed genes (DEGs) in the FFs compared to the FBs were enriched in several categories: terpenoid backbone biosynthesis, ubiquinone and other terpenoid-quinone biosynthesis, fatty acid metabolism, and flavonoid biosynthesis (arrows in Figure S9B). Expression analysis showed that most of the *TPS* genes were highly expressed in leaves and flowers in *J. sambac* (Figure 4A). We further performed qRT-PCR analysis on all 47 *TPS* genes to determine their relative expression levels in FBs, FFs, and normal leaves (NLs). Our results showed that 15 *TPS* genes (15/47, 31.91%) had higher expression levels in FBs than in FFs and NLs, whereas only 6 *TPS* genes (6/47, 12.77%) exhibited higher expression levels in FFs (Figure S10). Together with the qRT-PCR data, the results indicated that the mean expression level of *TPS* genes was higher in FBs than FFs (Figure 3F), suggesting that these genes may be involved in preparing the flower to release its fragrance. In addition, 26 *TPS* genes (26/47, 55.32%) showed higher expression levels in NLs than in FBs and FFs.

Terpenoids are the largest class of floral volatiles synthesized through MVA and 2-*C*-methylerythritol 4-phosphate (MEP) pathways. Therefore, we focused on the genes involved in terpene biosynthesis pathways and found that large numbers of putative *TPS* genes (38/47) were potentially involved in the synthesis of terpenes in both the MVA and MEP pathways (Figure 4A). Transcriptomic analysis revealed that many terpene biosynthesis-related genes were more highly expressed in FBs than in FFs, such as  3-hydroxy-3-methylglutaryl-coenzyme A reductase (*HMGR*) genes (4/10),  1-hydroxy-2-methyl-2-(E)-butenyl-4-diphosphate synthase (*HDS*) genes (2/2), and *TPS* genes (16/38). More importantly, some *TPS* genes encoding synthetases that regulate the synthesis of germacrene (sesquiterpene), geraniol (monoterpene), and α-terpineol (monoterpene) were also expressed at higher levels in FBs than in FFs, which may promote the synthesis of terpene products contributing to floral fragrance. However, RNA-seq and qRT-PCR analyses showed that three *TPS* genes encoding α-farnesene synthase (JS6G23500) and linalool synthases (JS4G16830 and JS4G13880), respectively, were highly expressed in FFs, and metabolomic analysis further revealed that α-farnesene and linalool contents were higher in FFs (Tables S13−S15). In addition, several other sesquiterpenes (such as isoledene and *cis*-caryophyllene) and diterpenes (muurolene) were also detected, all of which exhibited higher levels in FFs (Figure 4A).

**Supplementary Figure S11:**
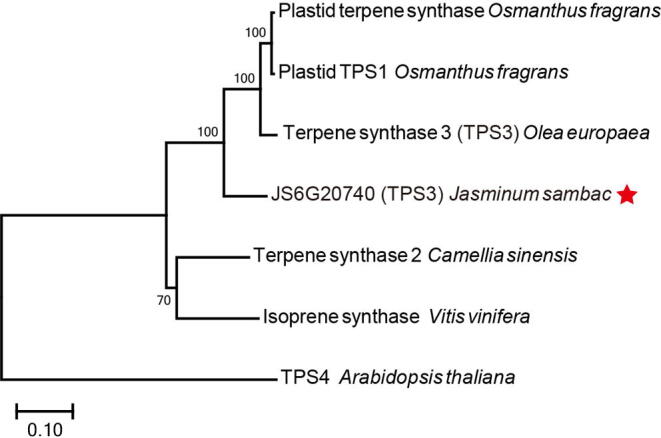
**Phylogenetic analysis of terpene synthase 3 in *J. sambac* homologs** JS6G20740 of *J. sambac* was annotated to terpene synthase 3 of *Olea europaea* (relative of *J. sambac* within the Oleaceae) based on protein–protein blastp in the NR database. Phylogenetic analysis also showed that JS6G20740 formed a well-supported clade as terpene synthase 3 of *Olea europaea*. Therefore, we considered JS6G20740 to be the TPS3 homolog, named JsTPS3.

**Supplementary Figure S12:**
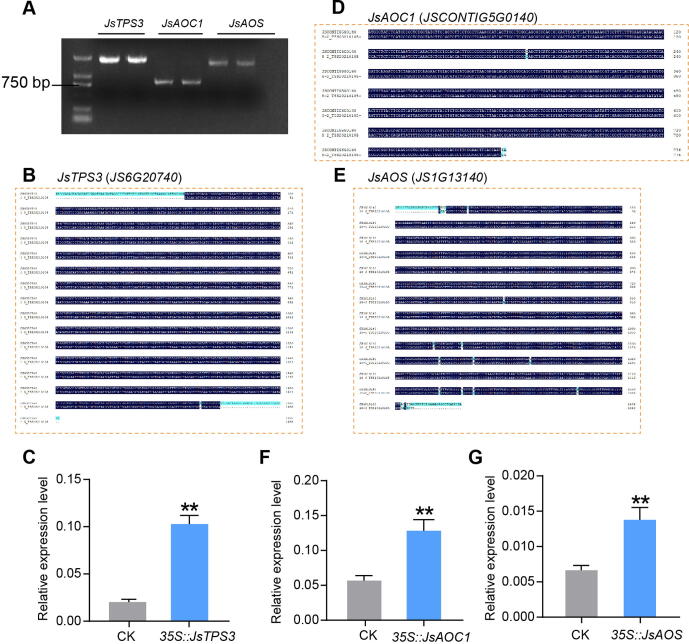
**Amplification and expression of *JsTPS3*, *JsAOC1*, and *JsAOS* genes from *J. sambac* leaves A.** The amplified bands of three genes. Coding regions of *JsTPS3* (**B**), *JsAOC1* (**D**), and *JsAOS* (**E**) of *J. sambac.* qRT-PCR confirmation of transgenic *J. sambac* callus overexpressing *JsTPS3* (C), *JsAOC1* (F), and *JsAOS* (**G**). Empty vector was used as a control. Bars are means ±SD from five biological replicates. **, *P* < 0.01 relative to control (Student’s *t*-test). CK, control; SD, standard deviation.

β-ocimene is one of the terpenoid constituents of *J. sambac* floral volatiles [10]. Based on the genome annotation as well as the phylogenetic and transcriptomic analyses of the TPS family, we found that *JsTPS3* (JS6G20740) encoding a putative β-ocimene synthase was significantly up-regulated in FFs and may regulate β-ocimene biosynthesis (Figure 4A, Figure S11). To validate the function of *JsTPS3*, we cloned the full-length CDS of *JsTPS3* from *J. sambac* (Figure S12A and B). We further constructed a *35S::pRI101-JsTPS3* plasmid and performed transient transformation in *J. sambac* calli. The expression level of *JsTPS3* increased significantly in calli, indicating successful transformation (Figure S12C). Furthermore, we detected two chiral β-ocimenes, *i.e.*, *trans*-β-ocimene and β-ocimene, which were significantly increased by 2.3- and 1.7-fold, respectively, in transformed *J. sambac* calli compared to controls (Figure 4B and C).

**Figure 5:**
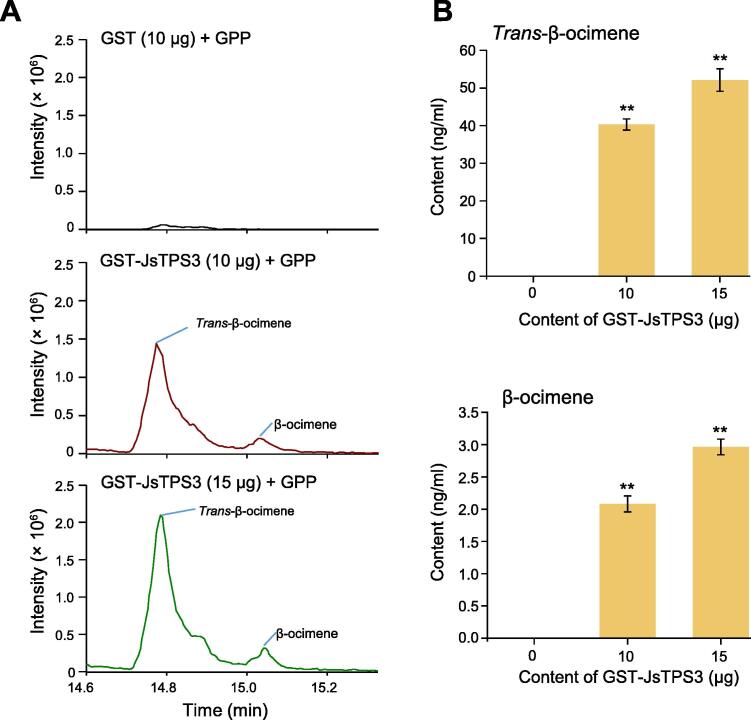
**JsTPS3 promotes β-ocimene biosynthesis validated by catalysisassays*in vitro*** **A.***In vitro* enzymatic products of recombinant JsTPS3 (GST-JsTPS3) proteins incubated with GPP (60 μM). The peak areas of *trans*-β-ocimene and β-ocimene in each enzymatic reaction are shown. *m/z* 136.1252 was used for EIC. **B.** Enzyme activity of purified GST-JsTPS3 in the presence of two concentrations of GST-JsTPS3 with GPP (60 μM) as the substrate. Data are presented as mean ± SD from three independent experiments. **, *P* < 0.01 (Student’s *t*-test). GST, glutathione *S*-transferase; EIC, extracted ion chromatography.

**Supplementary Figure S13:**
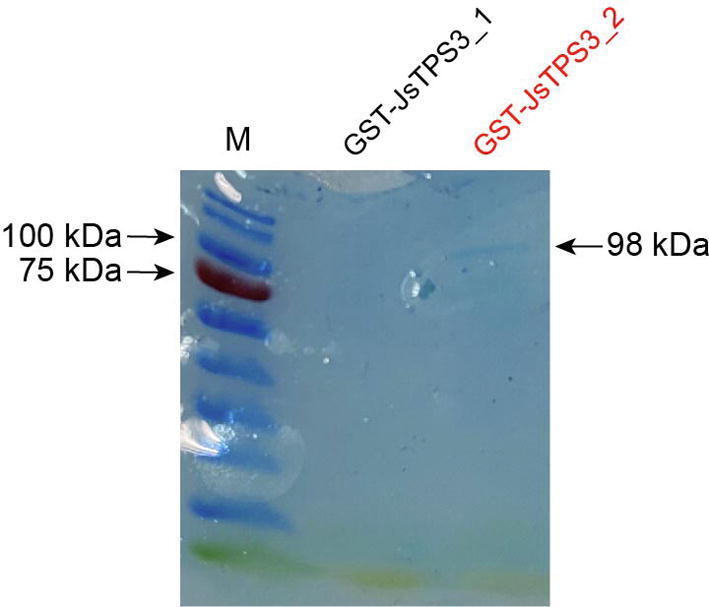
**Purification of GST-tagged JsTPS3 protein in *E. coli*** The gel was stained with Coomassie brilliant blue. SDS-PAGE results of recombinant JsTPS3 (two replicates) are shown. Lane 2 indicates the purified GST-JsTPS3 protein (∼98 kDa); no recombinant protein was detected in lane 1. M, molecular weight standards. SDS-PAGE, sodium dodecyl sulfate–polyacrylamide gel electrophoresis.

To further validate whether JsTPS3 is crucial for β-ocimene biosynthesis, we purified N-terminal glutathione *S*-transferase (GST)-tagged JsTPS3 protein (GST-JsTPS3) in an *Escherichia coli* expression system. We confirmed the purified GST-JsTPS3 protein by sodium dodecyl sulfate–polyacrylamide gel electrophoresis (SDS-PAGE; Figure S13). Moreover, we examined the enzyme activity of recombinant JsTPS3 *in vitro* (Figure 5A). We observed *trans*-β-ocimene and β-ocimene contents using different concentrations of GST-JsTPS3 with geranyl pyrophosphate (GPP) as the substrate, and relatively high TPS activity was measured with 15 μg of purified protein (Figure 5B). These results indicate that the recombinant JsTPS3 protein exerts TPS activity by catalyzing GPP into *trans*-β-ocimene and β-ocimene *in vitro*. These observations also demonstrate that JsTPS3 plays a vital role in the biosynthesis of β-ocimene, as an important component of the *J. sambac* floral scent.

### Phenylpropanoid/benzenoid pathway genes involved in the biosynthesis of volatile compounds

**Figure 6:**
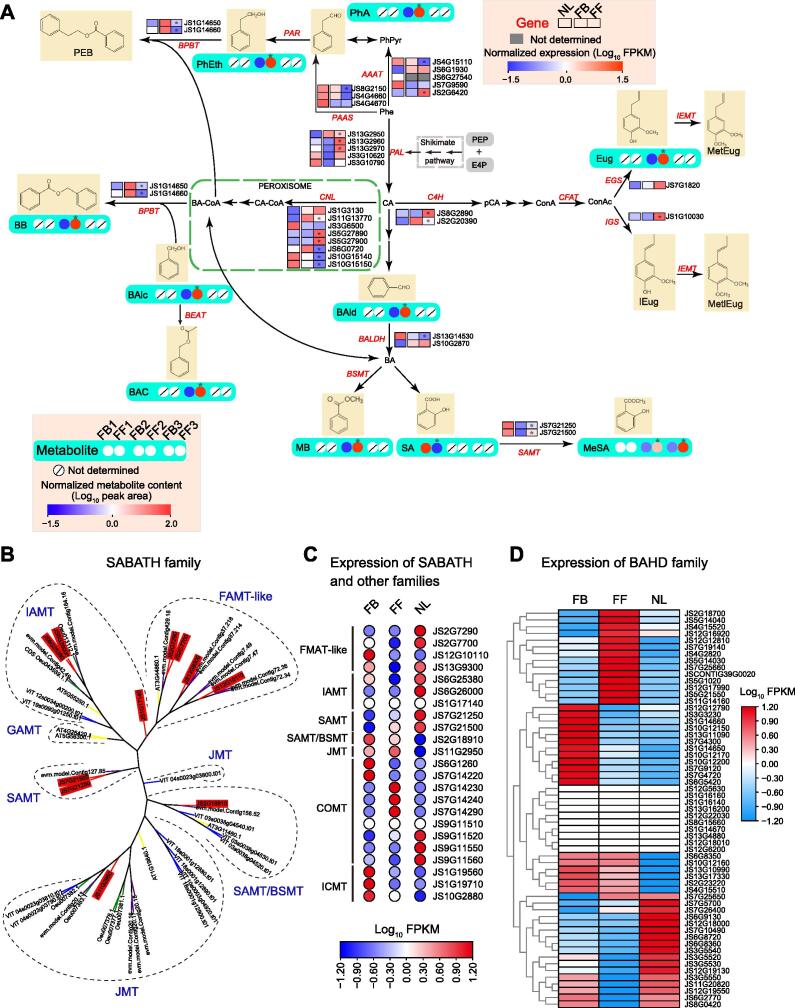
**Genes and metabolites involved inphenylpropanoid/benzenoid biosynthesis in *J. sambac*** **A.** Phenylpropanoid/benzenoid biosynthesis pathways in *J. sambac* leaves and flowers based on transcriptomic and metabolomic analyses. Metabolite contents in FB 1 *vs.* FF 1, FB 2 *vs.* FF 2, and FB 3 *vs.* FF 3 were determined by UPLC–ESI–MS/MS, GC–MS (metabolites exacted from flowers *in vitro*), and volatile GC–MS (volatiles released from flowers on living plants), respectively. Asterisks (*) indicate significant differences in gene exptession and metabolite content between FBs and FFs. DEGs were identified based on adjusted *P* < 0.05 and |log_2_ fold change| > 1. Differentially abundant metabolites were determined based on *P* < 0.05 and VIP ≥ 1 for UPLC–ESI–MS/MS and GC–MS, or FDR < 0.05 for volatile metabolites detected from flowers on living plants by GC–MS. The expression levels of genes and the contents of metabolites were normalized, with red and blue indicating high and low gene expression/metabolite content, respectively. **B.** Phylogenetic tree of the SABATH family in plants. The 11 SABATH homologs identified in *J. sambac* are shown in red backgrouds. **C.** Expression analysis of SABATH family genes and genes of other families in *J. sambac* leaves and flowers. **D.** Expression analysis of BAHD family genes in *J. sambac* leaves and flowers. SABATH, salicylic acid/benzoic acid/theobromine; BAHD, benzylalcohol *O*-acetyltransferase/anthocyanin *O*-hydroxycinnamoyltransferases/anthranilate *N*-hydroxycinnamoyl/benzoyltransferase/deacetylvindoline 4-*O*-acetyltransferase; Phe, phenylalanine; CA, cinnamic acid; PhPyr, phenylpyruvic acid; PhA, phenylacetaldehyde; PhEth, 2-phenylethanol; PEB, phenylethyl benzoate; CA-CoA, cinnamoyl-CoA; BA-CoA, benzoyl-CoA; BB, benzylbenzoate; BAld, benzaldehyde; BA, benzoic acid; MB, methylbenzoate; SA, salicylic acid; MeSA, methyl salicylate; BAlc, benzylalcohol; BAC, benzyl acetate; pCA, p-coumaric acid; ConA, coniferyl alcohol; ConAc, coniferyl acetate; Eug, eugenol; MetEug, methyl eugenol; IEug, isoeugenol; MetIEug, methyl isoeugenol; PAL, phenylalanine ammonia-lyase; AAAT, aromatic amino acid aminotransferase; PAR, phenylacetaldehyde reductase; BPBT, benzoyl-CoA:benzylalcohol/2-phenylethanol benzoyltransferase; PAAS, phenylacetaldehyde synthase; CNL, cinnamoyl-CoA ligase; C4H, cinnamate-4-hydroxylase; CFAT, coniferyl alcohol acetatyltransferase; EGS, eugenol synthase; IGS, isoeugenol synthase; IEMT, (iso)eugenol *O*-methyltransferase; BEAT, acetyl-CoA:benzylalcohol acetyltransferase; BALDH, benzaldehyde dehydrogenase; BSMT, benzoic acid/salicylic acid carboxyl methyltransferase; SAMT, salicylic acid carboxyl methyltransferase.

**Supplementary Figure S14:**
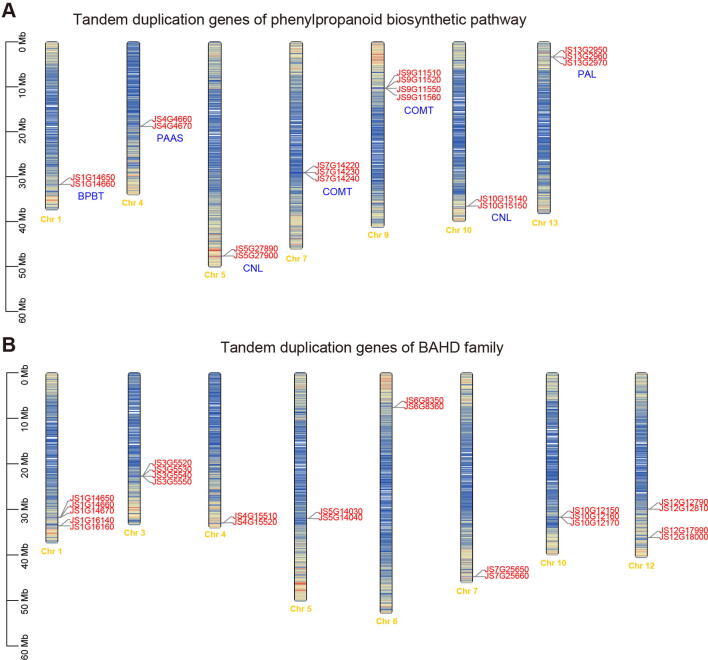
**Chromosomal distribution of the tandem duplication genes involved in the phenylpropanoid pathway in *J. sambac* A.** Tandem duplication of genes (red) involved in the phenylpropanoid biosynthetic pathway. **B.** Tandem duplication of genes (red) belonging to the BAHD family. BAHD, benzylalcohol O-acetyltransferase / anthocyanin O-hydroxycinnamoyltransferases / anthranilate N-hydroxycinnamoyl / benzoyltransferase / deacetylvindoline 4-O-acetyltransferase.

Phenylpropanoids and benzenoids represent the second largest class of flower VOCs [22] and are exclusively derived from the aromatic amino acid phenylalanine (Phe) (Figure 6A). Our metabolomic and transcriptomic analyses identified many genes (including 18 tandemly duplicated genes which were grouped into eight gene clusters distributed on different chromosomes; Figure S14A) and metabolites probably involved in the phenylpropanoid/benzenoid biosynthesis pathways (Figure 6A; Tables S16 and S17). The expression levels of the two genes (JS13G2960 and JS13G2970) encoding phenylalanine ammonia-lyase (PAL), the first committed enzyme in the phenylpropanoid/benzenoid pathways, were significantly higher in FFs than in FBs. Moreover, the expression levels of other genes, including aromatic amino acid aminotransferase (*AAAT*; JS2G6420), eugenol synthase (*EGS*; JS7G1820), isoeugenol synthase (*IGS*; JS1G10030), and salicylic acid carboxyl methyltransferase genes (*SAMTs*; JS7G21250 and JS7G21500), were also higher in FFs. Combined with the metabolomic analysis, we found that the contents of phenylacetaldehyde (PhA), eugenol (Eug), and methyl salicylate (MeSA), the downstream compounds catalyzed by AAAT, EGS, and SAMT, respectively, also increased significantly in FFs, indicating that these candidate genes may contribute to the accumulation of phenylpropanoid/benzenoid compounds (Figure 6A).

The production of phenylpropanoid/benzenoid compounds in plants is related to the salicylic acid/benzoic acid/theobromine (SABATH) and benzylalcohol *O*-acetyltransferase/anthocyanin *O*-hydroxycinnamoyltransferases/anthranilate *N*-hydroxycinnamoyl/benzoyltransferase/deacetylvindoline 4-*O*-acetyltransferase (BAHD) superfamilies. In our analyses, 11 SABATH homologs were identified, belonging to the IAMT (3), SAMT (2), JMT (1), SAMT/BSMT (1), and FAMT-like (4) subfamilies (Figure 6B). Transcriptomic analysis showed that the expression of three *FAMT-like* genes (JS2G7700, JS12G10110, and JS13G9300) and one *SAMT/BSMT* gene (JS2G18910) was higher in FBs than in FFs, whereas *JMT* and two *SAMT* genes (JS7G21250 and JS7G21500) were more highly expressed in FFs, and *IAMT* genes were expressed at low levels at both stages (Figure 6C). In addition, COMT and ICMT, belonging to the SAM-binding methyltransferase superfamily, are involved in aromatic compound metabolism [23]. Our results showed that the expression of *ICMT* genes was higher in FBs, whereas that of four *COMT* genes (JS7G14230, JS7G14240, JS7G14290, and JS9G11520) was higher in FFs (Figure 6C). Although the genes of the SABATH family showed different expression patterns between FBs and FFs, more of these genes were highly expressed in FBs than in FFs. BAHD acyltransferases are responsible for the synthesis of a myriad of flavors and fragrances in plants [22], [24], [25]. We identified a total of 59 *BAHD* genes (including 24 tandem duplicates which were grouped into 10 gene clusters; Figure S14B; Table S18) in *J. sambac*, and 17 *BAHD* genes were expressed at higher levels in FBs than in FFs and NLs (Figure 6D). The higher levels of expression of genes within the SABATH and BAHD superfamilies in FBs than in FFs suggests more active biosynthesis of volatile scent compounds in FBs than in FFs. Moreover, our metabolomic analysis revealed that most detected metabolites, including 2-phenylethanol (PhEth), benzylbenzoate (BB), benzylalcohol (BAlc), benzyl acetate (BAC), benzaldehyde (BAld), methylbenzoate (MB), and MeSA, accumulated significantly in FFs [*P* < 0.05, variable importance in projection (VIP) ≥ 1], whereas salicylic acid (SA) was higher in FBs (Figure 6A; Table S17). These metabolomic results indicate that large numbers of phenylpropanoid/benzenoid products are synthesized in FFs. In addition, the expression levels of most genes involved in phenylpropanoid/benzenoid biosynthesis (including SABATH and BAHD superfamilies) were significantly different between flowers (FBs and FFs) and leaves (NLs), indicating their different expression patterns in the phenylpropanoid/benzenoid pathways (Figure 6C and D).

### *JsAOC1* and *JsAOS* regulate jasmonate biosynthesis in *J. sambac*

**Figure 7:**
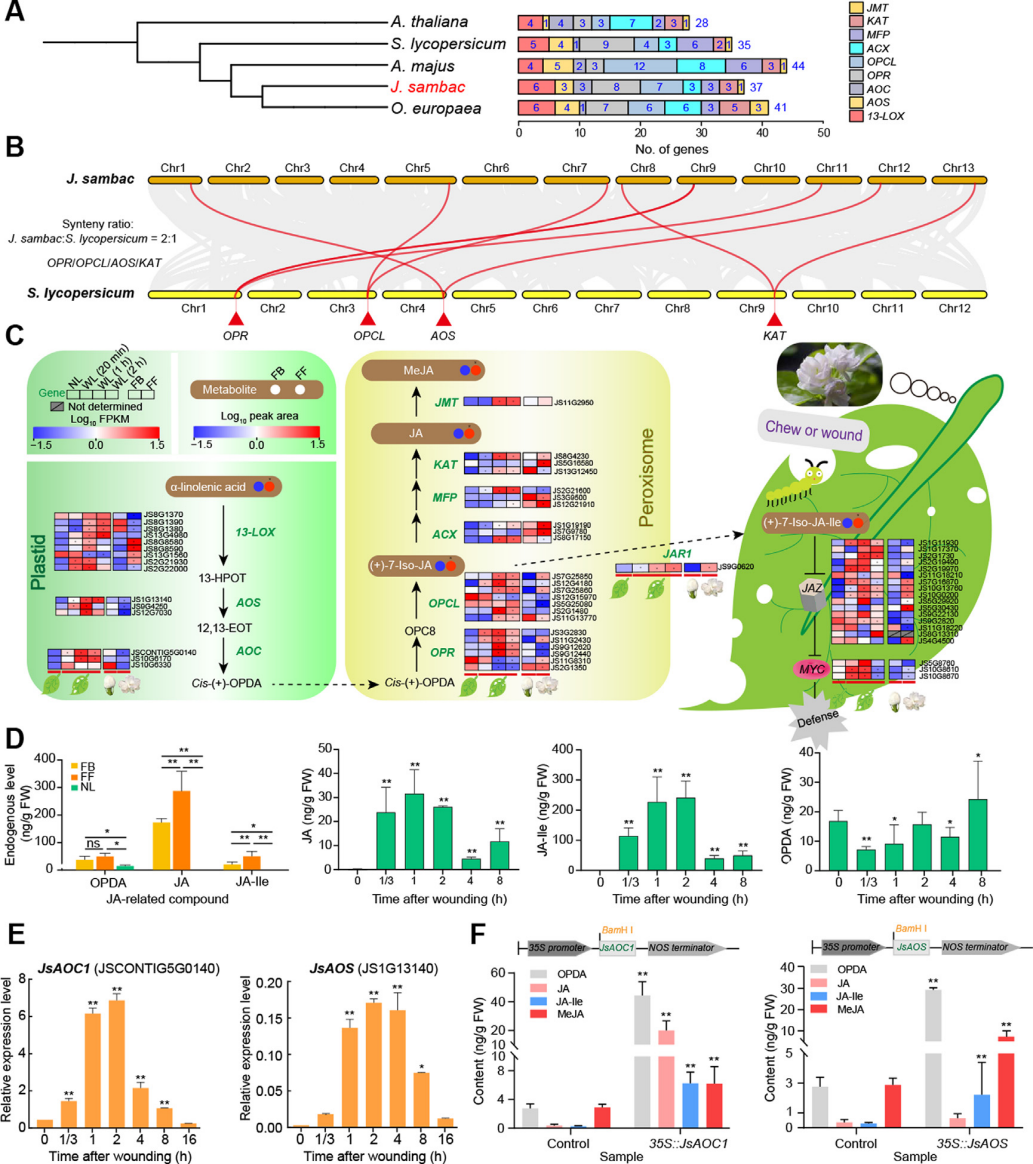
**Jasmonate contributes to flower scents and wounding response in *J. sambac*** **A.** Comparison of jasmonate biosynthesis-related genes in the genomes of *J. sambac*, *A. thaliana*, *S. lycopersicum*, *A. majus*, and *O. europaea*. **B.** Synteny analysis between genomes from *J. sambac* and *S. lycopersicum.***C.** Putative jasmonate biosynthesis pathways in *J. sambac*. The changes in gene expression and metabolite content among NLs, WLs, and flowers (FBs and FFs) were analyzed based on transcriptomic and metabolomic data. The expression levels of genes and the contents of metabolites were normalized, with red and blue indicating high and low gene expression/metabolite content, respectively. **D.** Contents of endogenous JA-related compounds in flowers (FBs and FFs) and WLs based on LC–MS. **E.** Expression of *JsAOC1* (JSCONTIG5G0140) and *JsAOS* (JS1G13140) in leaves at different time points after wounding by qRT-PCR. **F.** Contents of endogenous JA-related compounds in transgenic *J. sambac* calli. Data in (D−F) are shown as mean ± SD from at least three biological replicates. *, *P* < 0.05; **, *P* < 0.01 (one-way ANOVA with Dunnett’s post hoc test). WL, wounded leaf (followed by time after wounding); JA, jasmonic acid; MeJA, methyl jasmonate; JA-Ile, jasmonic acid-isoleucine; OPDA, 12-oxo-phytodienoic acid; OPR, OPDA reductase; OPCL, OPC-8:0 CoA ligase; AOS, allene oxide synthase; JMT, JA methyl transferase; KAT, 3-ketoacyl-CoA-thiolase; MFP, multifunctional protein; ACX, acyl-CoA-oxidase; AOC, allene oxide cyclase; 13-LOX, 13-lipoxygenase; JAZ, jasmonate ZIM-domain; MYC, myelocytomatosis; *A. majus*, *Antirrhinum majus*.

**Supplementary Figure S15:**
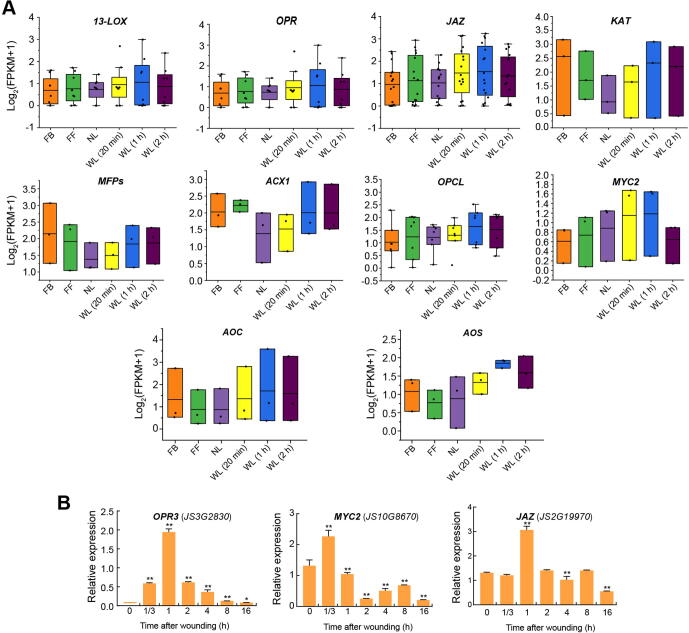
**Transcriptomicand qRT-PCR analysis of genes involved in jasmonate biosynthesis and JA signal transduction A.** Transcriptomic analysis of genes involved in jasmonate biosynthesis and JA signal transduction in normal leaves, wounded leaves, and flowers. **B.** qRT-PCR analysis of genes involved in jasmonate biosynthesis and JA signal transduction in leaves at different times after wounding. WL, wounded leaves (followed by time after wounding); JA, jasmonic acid; FPKM, fragments per kilobase million.

The jasmonic acid (JA) biosynthesis pathway involves multiple genes encoding enzymes, including 13-lipoxygenase (13-LOX), allene oxide synthase (AOS), allene oxide cyclase (AOC), 12-oxo-phytodienoic acid (OPDA) reductase (OPR), OPC-8:0 CoA ligase (OPCL), acyl-CoA-oxidase (ACX), multifunctional protein (MFP), 3-ketoacyl-CoA-thiolase (KAT), and JA methyl transferase (JMT) [26]. We identified a total of 37 JA biosynthesis-related genes in *J. sambac* using bidirectional BLAST hits and conserved domain examination (Table S19). Based on comparisons with the genomes of *A. thaliana* (28), *S. lycopersicum* (35), *Antirrhinum majus* (44), and *O. europaea* (41), no obvious expansion of the JA biosynthesis-related genes was found in the *J. sambac* genome. As the JA biosynthesis and signaling pathways have been clearly elucidated in *A. thaliana*, we compared the JA biosynthesis-related genes in *J. sambac* with those in *A. thaliana*. The results indicate that the *J. sambac* genome has more JA biosynthesis-related genes than *A. thaliana* (Figure 7A). Several key genes involved in the regulation of JA biosynthesis, including *OPR*, *OPCL*, *AOS*, and *KAT*, were present at a ratio of 2:1 relative to their presence in the tomato genome, indicating duplication of these genes in *J. sambac* resulting from the Oleaceae-specific WGD event (Figure 7B). Transcriptomic analysis revealed that the expression levels of JA biosynthesis-related genes in FBs and FFs were quite different. The expression levels of genes in the JA biosynthesis pathway, especially *AOSs* (JS1G13140, JS9G4250, and JS12G7030), *AOC1* (JSCONTIG5G0140), *MFPs* (JS2G21600 and JS3G9500), and *KAT* (JS13G12450), were much higher in FBs than in FFs, whereas those of β-oxidation genes, such as *OPRs* (JS11G2430, JS11G8310, and JS2G1350), *OPCLs* (JS7G25850, JS12G4180, and JS7G25860), and *ACXs* (JS1G19190 and JS7G9780), were higher in FFs (Figure 7C, Figure S15A). In the signaling pathway, the expression levels of *TIFY6B* (JS11G18210) and *TIFY4B* (JS11G18220) were higher in FBs than in FFs, whereas those of *TIFY10B* (JS10G0200), *TIFY6A* (JS5G30430), and *TIFY5A* (JS4G4500) were higher in FFs (Figure 7C). In addition, metabolomic and liquid chromatography–mass spectrometry (LC–MS) analyses both showed that jasmonates were enriched in FFs and FBs. The contents of JA, methyl jasmonate (MeJA), and jasmonic acid-isoleucine (JA-Ile), as well as the content of their precursor α-linolenic acid, were higher in FFs than in FBs (Figure 7C and D; Table S16). Moreover, LC–MS analysis also revealed that JA and JA-Ile contents were considerably higher in leaves at 20 min to 2 h after wounding, while the content of their intermediate metabolite OPDA was considerably higher at 8 h after wounding (Figure 7D). Interestingly, some genes with high expression in flowers had low expression in leaves, but these genes were significantly highly expressed in wounded leaves (WLs; Figure 7C, Figure S15A). Specifically, the mean expression levels of JA biosynthesis-related genes [*AOSs* (JS1G13140, JS9G4250, and JS12G7030), *ACX1* (JS1G19190, JS7G9780, and JS8G17150), and *KATs* (JS8G4230, JS5G16580, and JS13G12450)] were higher in WLs than in NLs, especially at 1–2 h after wounding, and JA signal transduction-related genes [*JAZ* (JS2G19970) and *MYC2* (JS10G8670)] were activated at 20 min to 1 h after wounding (Figure S15A). qRT-PCR analysis also demonstrated that the expression of JA biosynthesis-related genes [*AOC1* (JSCONTIG5G0140), *AOS* (JS1G13140), and *OPR3* (JS3G2830)] and JA signal transduction-related genes [*JAZ* (JS2G19970) and *MYC2* (JS10G8670)] were considerably increased in leaves at 20 min after wounding (Figure 7E, Figure S15B). Of note, the expression levels of JS5G30430 (one of the *JAZ* genes) were low in both normal and wounded leaves. However, it was highly expressed in FFs (Figure 7C), indicating that JS5G30430 mainly responds to endogenous signals during flower development rather than participating in the JA-mediated injury response.

The results of our multi-omics and qRT-PCR analyses indicated that the expression of *JsAOC1* (JSCONTIG5G0140) and *JsAOS* (JS1G13140) was highly up-regulated at 1 h and 2 h after leaf wounding (Figure 7E, Figure S15A). Further analyses of the contents of JA compounds showed that JA and JA-Ile levels were also significantly increased (Figure 7D) after wounding. Based on these results, we speculated that *JsAOC1* and *JsAOS* may play important roles in JA biosynthesis in *J. sambac*. To validate their functions, we cloned the full-length CDS of *JsAOC1* (JSCONTIG5G0140) and *JsAOS* (JS1G13140), and then constructed *35S::pRI101-JsAOC1* and *35S::pRI101-JsAOS* plasmids, respectively (Figure S12A, D, and E). The levels of *JsAOC1* and *JsAOS* expression were significantly increased in *J. sambac* calli after transformation with these plasmids (Figure S12F and G). Furthermore, we measured the contents of OPDA and JA and found that they were significantly increased in the transformed *J. sambac* calli compared to controls (Figure 7F). These results indicated that both *JsAOC1* and *JsAOS* are involved in the biosynthesis of JAs in *J. sambac*.

## Discussion

*J. sambac* flowers are widely used in ornamental horticulture, perfume industry, scented tea, food, and pharmaceutical applications [2], [27], [28]. In this study, we sequenced and assembled a chromosome-level genome (550.12 Mb) of *J. sambac* with a scaffold N50 length of 40.10 Mb; 97.36% of the genome sequences were anchored onto 13 pseudochromosomes; and BUSCO evaluation revealed that the genome covers 97.20% of the complete orthologs of plant-specific sequences. These results indicate that our genome assembly is precise, complete, and of high quality. In addition, 30,129 genes were annotated, 93.20% of which had predicted functions, indicating high annotation quality. This high-quality genome assembly of *J. sambac* provides a fundamental genetic resource for functional genomic research and understanding of fragrance biosynthesis mechanisms in *J. sambac*.

In the Oleaceae family, the genomes of five species have been sequenced, including *F. excelsior* [29], *O. europaea* [30], *O. oleaster* [31], *O. fragrans* [32], and *Forsythia suspense* [33]. Among them, only *O. fragrans* flowers are scented with a sweet aroma, with VOCs including linalool, dihydrojasmone lactone, 1-cyclohexene-1-propanol, and β-ocimene [34]. In contrast, the aroma type of *J. sambac* flowers is considerably different. During the flowering period, as their petals fully expand, the corolla tube in FFs becomes markedly elongated compared to that in FBs (Figure 1C and D), indicating that this developmental stage is for flower fragrance release. We identified the major volatile fragrance compounds in *J. sambac* flowers as terpenoids (linalool, γ-muurolene, isoledene, and farnesene), phenylpropanoids/benzenoids (PhA, PhEth, BB, BAlc, BAC, and MeSA), and fatty acids (α-linolenic acid, JA, and MeJA) (Tables S14 and S16). These VOCs seem to be responsible for the unique scent of *J. sambac* flowers. Generally, plant VOC biosynthesis is regulated by many genes and gene families [22], [35]. For example, the terpenoids are biosynthesized via TPS-dependent pathways [35], [36]. In the genome of *J. sambac*, we identified many *TPS* genes present as gene clusters through recent tandem duplications, resulting in considerably amplified *TPS* genes in the genome. Therefore, these *TPS* gene clusters likely contribute to the terpenoid fragrance of *J. sambac* flowers. Moreover, the terpenoid fragrance that evaporates into the air differs significantly between FBs and FFs. This can be explained by the differential expression of *TPS* genes between these two stages. Particularly, our results regarding transient transformation and enzymatic activity experiments confirmed the important roles of *JsTPS3* in the β-ocimene biosynthesis of *J. sambac*. In addition, FF petal cells are plump, with larger intercellular spaces, compared to those in FBs (Figure 1E and F), suggesting that the separated petal cells increase the emission of fragrant compounds in FFs. These morphological, transcriptomic, and metabolomic analyses collectively suggest that fragrance release from *J. sambac* flowers is a dynamic stage-dependent process.

Volatile phenylpropanoids and benzenoids are major volatile aroma compounds present in plants [10]. They originate from the aromatic amino acid Phe. Several other antioxidant metabolites are also synthesized from Phe, including flavonoids and anthocyanin pigments [37], [38]. Phe is deaminated to cinnamic acid (CA) by PAL. CA is further converted into diverse volatile compounds via three main synthetic routes of enzymatic and acid-catalyzed transformations: BB, catalyzed by cinnamate-coenzyme A ligase (CNL); Eug, catalyzed by EGS; and MB, SA, and MeSA, catalyzed by benzaldehyde dehydrogenase (BALDH). Phe can also be converted to phenylethyl benzoate (PEB) via PhA and PhEth, catalyzed by PAR and BPBT, respectively [22]. In our analysis, these phenylpropanoid/benzenoid volatile compounds, including BB, Eug, MB, MeSA, PhA, and PhEth (Table S17), showed greater accumulation in FFs than in FBs, whereas SA was detected at higher levels in FBs (Figure 6A). Furthermore, many other metabolites in the phenylpropanoid/benzenoid pathways were also detected in our analysis, such as phenylpyruvic acid, ferulic acid, 2,3-dihydroxybenzoic acid, and benzoic acid. These volatile compounds also contribute to the specific aroma of *J. sambac* flowers. Expression analysis of the phenylpropanoid/benzenoid pathway genes by RNA-seq revealed that *PALs* (JS13G2960 and JS13G2970), *AAAT* (JS2G6420), *EGS* (JS7G1820), *IGS* (JS1G10030), and *SAMTs* (JS7G21250 and JS7G21500) were highly expressed in FFs, whereas *BPBTs* (JS1G14650 and JS1G14660) and *CNLs* (JS11G13770, JS6G0720, JS10G15140, and JS10G15150) were more highly expressed in FBs. Apparently, the regulation of gene expression at varying stages coordinates VOC dynamics during the timeline of flower blooming. Notably, we identified many flavonoids (38 of 174) that were enriched in *J. sambac* flowers. As flavonoids are important secondary metabolites with antioxidant properties, and jasmine tea is a common beverage, the jasmine flowers in tea may be beneficial to human health in addition to providing aroma.

Fatty acids and their derivatives represent another important type of fragrant VOCs in *J. sambac* flowers. Among them, jasmonate and its related compounds, including JA, MeJA, JA-Ile, and jasmone, are fragrant components of the essential oils of jasmine flowers [2], [13]. In our metabolomic analyses, these jasmonate-related compounds were enriched in *J. sambac* flowers, indicating their important roles in the formation of the characteristic aromatic odor of the flowers. In addition, JA, MeJA, and JA-Ile play important roles in plant defense against biotic and abiotic stresses [18], [39]. Our analysis revealed that the JA contents and related genes in *J. sambac* leaves responded to mechanical injury. For example, several genes [*e.g.*, *AOC1* (JSCONTIG5G0140) and *AOS* (JS1G13140)] involved in the JA biosynthesis pathway were highly expressed in WLs and the JA signal transduction-related genes [*JAZ* (JS2G19970) and *MYC2* (JS10G8670)] were also activated after wounding. These results were further confirmed by qRT-PCR and transient transformation. Compared to *Arabidopsis* and tobacco [40], [41], the response times of genes involved in JA signaling and biosynthesis pathways to wounding are similar in leaves, suggesting a similar responsive pattern in *J. sambac.* However, in the JA signaling pathway, the expression levels of some *JAZ* genes, such as JS11G18210 and JS11G18220, were significantly higher in FBs, whereas those of other *JAZ* genes (JS7G16870, JS10G13760, JS10G0200, JS5G30430, and JS4G4500) were higher in FFs, suggesting significant differences in the functions of the *JAZs* involved in flower development of *J. sambac*. Moreover, *JAZs* and *MYCs* were significantly activated with the high JA content in FFs, suggesting that flowering is an important period for biosynthesis of jasmonates and JA-related floral aroma substances. These findings demonstrated that regulation of the JA signaling pathway during *J. sambac* flower development is related to robust secondary metabolism. In particular, MeJA has been reported to induce the expression of terpenoid- and phenylpropanoid/benzenoid-related genes and promote the synthesis of related metabolites [37], [42]. Therefore, these jasmonates in blooming *J. sambac* flowers may also affect the synthesis and release of other floral aromatic components. As an important aroma itself, MeJA together with other aromatic components may orchestrate the unique sweet fragrance of *J. sambac* flowers. However, other than attracting pollinators, the further biological significance of jasmonates in *J. sambac* flowers requires additional research.

**Figure 8:**
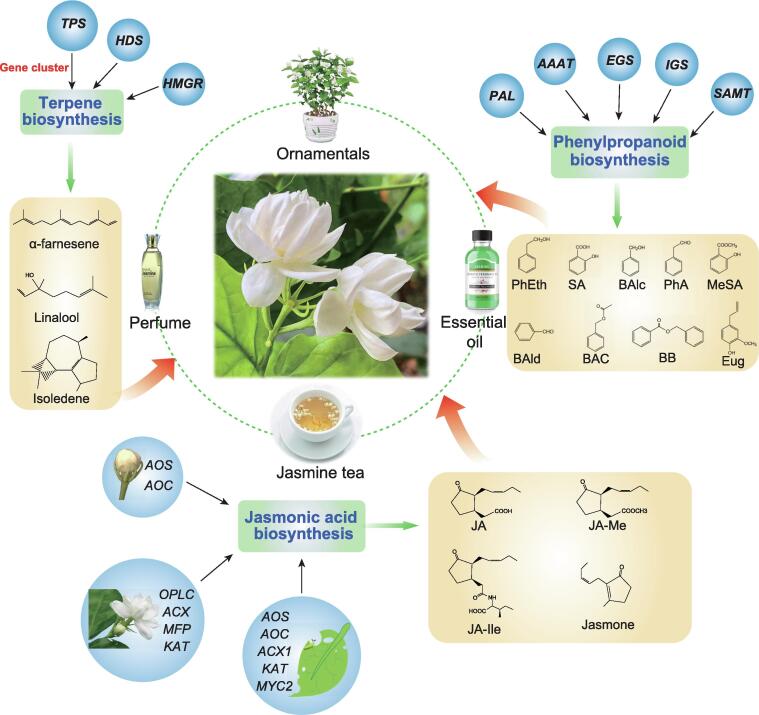
**Schematic diagram of genes, compounds, and applications in *J. sambac*** Genes involved in terpene, phenylpropanoid, and jasmonic acid biosyntheses contribute to the production of the aromatic compounds in *J. sambac* flowers and their commercial applications of *J. sambac*.

In summary, we presented a chromosome-level genome of *J. sambac* and identified the main volatile aroma compounds in *J. sambac* FBs and FFs. Our multi-omics analyses revealed the transcriptomic and metabolomic profiles of jasmine volatile aroma production (Figure 8). This high-quality, annotated genome sequence of *J. sambac* together with the transcriptomic and metabolomic datasets in this study provides a fundamental genetic resource for studying functional genomics and fragrance biosynthesis in *J. sambac*, which will be invaluable for the industrial exploitation of jasmine flowers in the future.

## Materials and methods

### Plant materials for genome sequencing

The *J. sambac* ‘double petal’ cultivar, the major cultivar in China, was selected as the model plant species for studying jasmine flowers (Figure 1). All materials (∼ 20 plants) were sampled from individual potted plant clones with the same genetic background in the greenhouse of Yangzhou University, Yangzhou, China (32.39°N, 119.42°E). The newly expanded leaves from the sequenced plant were disinfected with 70% ethyl alcohol and rinsed with distilled water. They were then harvested and immediately frozen in liquid nitrogen and stored at −80 °C prior to DNA extraction.

### Estimation of the genome size

To estimate the *J. sambac* genome size, *k*-mer analysis with Illumina sequencing short reads (89.05 Gb, sequencing depth of 162×) was performed. A *k*-mer refers to an oligonucleotide *k*-bp in length. The *k*-mer frequencies derived from the sequencing reads follow a Poisson distribution in a given dataset (Figure S1). Based on the obtained sequencing data, the sequence fragments of length *k* were iteratively selected. The total number of *k*-mers (*n*) was calculated using the formula *n* = *l* − *k* + 1, where *l* is the length of each sequence analyzed, and *k* is the selected length of *k*-mer. After all *k*-mer sequences have been obtained, the genome size can be estimated based on the frequency of *k*-mer sequences. The genome size was calculated using the formula *n*/*d*, where *n* is the total number of *k*-mers and *d* is the coverage depth. In our survey analysis, *k* = 17 was selected for the estimation of *J. sambac* genome size, and the number of *k*-mers (*n*) and coverage depth (*d*) were 64,963,845,770 and ∼ 112 (main peak value), respectively (Figure S1). Finally, we estimated that the genome size of *J. sambac* was ∼ 580.03 Mb (64,963,845,770/112) with *k*-mer methods using Jellyfish (version 2.1.3) [43], and after removing the wrong *k*-mers, the corrected genome size was ∼ 573.02 Mb.

### PacBio library construction and sequencing

Genomic DNA was extracted by the CTAB method [44], and at least 10 μg of sheared DNA was obtained. SMRT bell template preparation, including DNA concentration, damage repair, end repair, ligation of hairpin adapters, and template purification, was conducted using AMPure PB magnetic beads (Catalog No. 100-265-900, PacBio, Menlo Park, CA). We conducted 20-kb SMRT DNA sequencing using PacBio to sequence a DNA library on the PacBio Sequel platform, and 63.90 Gb of raw sequencing reads were obtained. Reads were trimmed for adapter removal and quality enhancement, yielding 63.82 Gb of PacBio data (read quality ≥ 0.80, mean read length ≥ 14 kb) representing 116× genome coverage.

### *De novo* genome assembly

Before *de novo* assembly, low-quality PacBio subreads with a read length < 500 bp or a quality score < 0.8 were filtered out. The remaining clean PacBio subreads were error-corrected (self-correction) and pre-assembled into contigs using FALCON software (version 1.2.4; with the parameters: “overlap_filtering_setting = --max_diff 500 --max_cov 500 --min_cov 2 --n_core 20”) [45]. The assembled scaffolds were polished with Quiver in SMRT analysis (SMRT Link v5.0.1, https://pbsmrtpipe.readthedocs.io/en/master/getting_started.html; with the default parameters) in two rounds. Then, the short reads from Illumina were used to correct any remaining errors by Pilon (version 1.22; with the default parameters) [46] in two rounds. The heterozygosity of assembly was removed by using the Purge Haplotigs software [47], accordingly, a draft genome assembly of *J. sambac* was generated.

### Pseudochromosome validation using Hi-C

To avoid artificial bias, the following reads were removed: reads with ≥ 10% unidentified nucleotides (Ns); reads with > 10 bp aligned to the adapter, allowing ≤ 10% mismatches; and reads with > 50% bases having Phred quality < 5. The filtered Hi-C reads were aligned to the initial pseudochromosome genome using BWA (version 0.7.8) [48] with the default parameters. Reads were excluded from subsequent analysis if they did not align within 500 bp of a restriction site. Only paired-end reads that were uniquely mapped with valid ditags were used to validate the pseudochromosome sequences. Juicebox (https://github.com/aidenlab/Juicebox; with the default parameters) was used to manually order the scaffolds in each group to obtain the final pseudochromosome assembly. Contact maps were plotted using HiCPlotter (https://github.com/kcakdemir/HiCPlotter) with the default parameters.

### Assessment of genome quality

The completeness and accuracy of the *J. sambac* assembly were evaluated by multiple methods. First, the 1614 conserved protein models in the BUSCO “embryophyta_odb10” dataset were queried against the *J. sambac* genome using the BUSCO (version 4.0.2) [19] program with the default settings (Table S4), and we obtained a genome completeness value of 97.20%. LAI (https://github.com/oushujun/LTR_retriever) and CEGMA [49] evaluations were conducted to assess the completeness of genome assembly. For LAI estimation, the LAI value was obtained from the outputs of LTR_RETRIEVER [20], and its estimated value ≥ 20 was considered the gold standard. For CEGMA, the conserved genes (248 core eukaryotic genes) from six eukaryotic model organisms were used to construct the core gene library, and then the estimation was performed using CEGMA (version 2.5). To assess the accuracy of genome assembly, the DNA-seq and RNA-seq reads were aligned to the assembly using BWA software, and the mapping rate or genome coverage of reads was analyzed.

### Repeat annotation

Repeat elements in the *J. sambac* genome were annotated using a combined strategy based on sequence homology and *de novo* prediction. Tandem repeats were also identified by tandem repeats finder (https://tandem.bu.edu/trf/trf.html). The homology-based BLAST was performed against the Repbase database (https://www.girinst.org/repbase), and then RepeatProteinMask searches (https://www.repeatmasker.org/) were performed for the prediction of homologs [50]. For *de novo* annotation, LTR_FINDER (https://tlife.fudan.edu.cn/tlife/ltr_finder/; with the parameters: “-C -w 2”) [51], Piler (https://www.drive5.com/piler/; with the default parameters) [52], RepeatScout (with the parameters: “-L 10000 -maxgap 5”) [53], and RepeatModeler (version 1.0.5, https://www.repeatmasker.org/RepeatModeler/; with the parameters: “-engine ncbi”) were used to construct a *de novo*-based repeat library. The non-redundancy repeat library was finally used to predict repeat elements using RepeatMasker (version 3.3.0; with the parameters: “-a -nolow -no_is -norna”) [54].

### RNA-seq-based prediction for gene annotation

To aid gene annotation and perform the transcriptomic analysis, RNA was extracted from six tissues (roots, shoots, adult NLs, WLs, FBs, and FFs) of *J. sambac*. All fresh tissues were first frozen in liquid nitrogen and stored at −80 °C before processing. Total RNA of each sample was extracted using TRIzol Reagent (Catalog No. 15596026, ThermoFisher Scientific, Waltham, MA) according to the manufacturer’s instructions and mixed together. RNA-seq libraries were prepared using the Illumina standard mRNA-seq library preparation kit and sequenced on the Illumina HiSeq 4000 platform using a paired-end sequencing strategy. A full-length isoform sequencing (ISO-seq) library was also constructed with an insert size of 0–5 kb using the same samples, then sequenced on the PacBio SMRT Sequel platform at Novogene (Tianjin, China). The ISO-seq reads were extracted using the SMRT Link (version 7.0; https://www.pacb.com/support/software-downloads/) software with the default parameters, to obtain the polished consensus sequences; these data were further processed by the CD-hit software (with the default parameters) to remove redundancies. In addition, the completeness of gene annotation of *J. sambac* was assessed using BUSCO with “embryophyta_odb10” dataset [19].

### Annotation of protein-coding genes

Protein-coding genes were annotated using a comprehensive strategy integrating results obtained from homology-based prediction, *de novo* prediction, and RNA-seq-based prediction methods. Annotated protein sequences from *F. excelsior*, *O. fragrans*, *O. europaea*, and *O. europaea* var. *sylvestris* (Oleaceae family) were aligned to the *J. sambac* genome assembly using WU-BLAST (https://blast.wustl.edu/) [55] with an E-value cutoff of 1E−5, and the hits were conjoined using the Solar software (version 0.0.19) [56]. GeneWise (version 2.2.0) [57] was used to predict the exact gene structure of the corresponding genomic regions for each WU-BLAST hit. The gene structure created by GeneWise was denoted as the homology-based prediction gene set (Homo-set). Gene models created by Program to Assemble Spliced Alignments (PASA; https://github.com/PASApipeline/PASApipeline/wiki) were denoted as the PASA ISO-seq set (PASA-ISO-set), and were used as the training data for the *de novo* gene prediction programs. Five *de novo* gene-prediction programs [Augustus (version 3.0.2), GENSCAN (version 1.0), GeneID, GlimmerHMM (version 3.0.2), and SNAP] [58], [59], [60], [61], [62] were used to predict coding regions in the repeat-masked genome. Illumina RNA-seq data were mapped to the assembly using TopHat [63] (with the parameters “-N 2 --read-gap-length 2”), and then Cufflinks [64] (with the parameters “-m 200 -s 80”) was used to assemble the transcripts into gene models (Cufflinks-set). In addition, RNA-seq data were assembled by Trinity [65] (with the parameters “--min_glue 2 --min_kmer_cov 2”), creating several pseudo-expressed sequence tags (ESTs). These pseudo-ESTs were also mapped to the assembly by LASTZ (https://github.com/lastz/lastz/) with the default parameters, and gene models were predicted using PASA with the default parameters. PacBio ISO-seq sequences were mapped directly to the *J. sambac* genome assembly by BLAT [66] (with the parameters “-maxGap 2 -maxIntron 750000”) and assembled by PASA. This gene set was denoted as the PASA Trinity set (PASA-T-set). Gene model evidence data from the Homo-set, PASA-ISO-set, Cufflinks-set, PASA-T-set, and *de novo* programs were combined by EvidenceModeler [67] into a non-redundant set of gene annotations. Weights for each type of evidence were set as follows: PASA-ISO-set > Homo-set > PASA-T-set > Cufflinks-set > Augustus > GeneID = SNAP = GlimmerHMM = GENSCAN. Gene models with low-confidence scores were filtered out by the following criteria: (1) coding region lengths of ≤ 150 bp, (2) supported only by *de novo* methods and with FPKM < 1.

All protein-coding genes were aligned to two integrated protein sequence databases: Swiss-Prot and NR. Protein domains were annotated by searching against InterPro database (version 32.0) using InterProScan (version 4.7) [68] and against the Pfam database (version 27.0) using HMMER (version 3.0) [69]. The GO terms for each gene were obtained from the corresponding InterPro or Pfam entry. The pathways in which the genes might be involved were assigned by BLAST searches against the KEGG database, with an E-value cutoff of 1E−5. Functional annotation results from the three strategies mentioned above were finally merged. The annotation results can be found in Table S6. In total, 30,129 genes were predicted to be functional, accounting for 93.2% of all genes in the *J. sambac* genome (Table S7).

### Annotation of non-coding RNAs

The tRNAs were annotated using tRNAscan-SE (https://lowelab.ucsc.edu/tRNAscan-SE/; with the parameters: “-X 20 -z 8”) [70]. The miRNAs and small nuclear RNAs were predicted using INFERNAL (https://infernal.janelia.org/; with the default parameters) [71] with searches against the Rfam database (version 14.1). Because rRNA sequences are highly conserved among plants, rRNAs from *A. thaliana* were screened by BLAST searches with E-value ≤ 1E−5 (Table S9).

### Comparative genomic analysis

Orthologous relationships between genes of *J. sambac*, *O. fragrans*, *F. excelsior*, *O. europaea*, *O. oleaster*, *Prunus mume*, *Petunia inflata*, *C. sinensis*, *Populus trichocarpa*, *S. lycopersicum*, *V*. *vinifera*, *Medicago truncatula*, *Oryza sativa*, *A. thaliana*, *A. majus*, *Amborella trichopoda*, and *Salvia splendens* were inferred through all-against-all protein sequence similarity searches using OrthoMCL (https://orthomcl.org/orthomcl/); only the longest predicted transcript per locus was retained. The paralogous genes within *J. sambac* were identified by OrthoMCL. For all single-copy orthologous genes, multiple sequence alignment was performed by MUSCLE (https://www.drive5.com/muscle/; with the parameter “-maxiters 16”), and the ambiguously aligned positions were trimmed using Gblocks (https://molevol.cmima.csic.es/castresana/Gblocks.html; with the default parameters). Finally, the phylogenetic tree was constructed using RaxML (version 7.2.9, https://sco.h-its.org/exelixis/software.html; with the parameter “-p 12345 -x 12345”). Divergence time between the aforementioned species was inferred using Bayesian Markov-chain Monte Carlo tree (MCMCTree; “seed = -1”) package implemented in phylogenetic analysis by maximum likelihood (PAML; version 4.7) [72] with the main parameters of “burn-in = 100,000, sample-number = 100,000, and sample-frequency = 2”. Eight calibration points were selected to refer to the published literature [73], [74] and from the TimeTree database (https://www.timetree.org/; used as normal priors) [75] to confine the ages of nodes. The detailed calibration points are as follows: *A. thaliana*–*P. mume* (107–109 MYA), *A. thaliana*–*V. vinifera* (105–115 MYA), *A. thaliana*–*C. sinensis* (112–125 MYA), *A. thaliana*–*O. sativa* (171–203 MYA), *O. europaea*–*F. excelsior* (36–44 MYA), *O. europaea*–*S. lycopersicum* (72–89 MYA), *A. majus*–*A. splendens* (56–76 MYA), and the crown node of angiosperms (234–263 MYA).

### Expansion and contraction of gene families

To identify gene family evolution as a stochastic birth and death process in which a gene family either expands or contracts per gene per million years independently along each branch of the phylogenetic tree, we used the likelihood model originally implemented in the software package Café (https://sourceforge.net/projects/cafehahnlab/). The topology and branch lengths of the phylogenetic tree were taken into account to infer the significance of changes in gene family size in each branch.

### WGD analysis

We applied *Ks* estimation and syntenic comparison to detect WGD events. First, the protein sequences from *J. sambac*, *O. fragrans*, and *V. vinifera* were aligned against within and between species using BLASTp (E-value ≤ 1E−7). Next, the synteny blocks within a genome or between genomes were identified with the detected homologous gene pairs by McscanX [76]. For each gene pair in a syntenic block, the *Ks* value was calculated with the codeml program in PAML (version 4.7) [72]. The base substitution rate of jasmine was calculated as *r* = 1–1.3 × 10^−8^ according to the previous method by Badouin and colleagues [77]. Thus, the time of WGD was determined by *T* = *Ks*/2*r*. For multiple genome syntenic comparisons and visualizations, the JCVI (version 1.2.7) package (https://github.com/tanghaibao/jcvi) was used.

### Copy number analysis of *TPS*, *SABATH*, *BAHD*, and JA biosynthesis-related genes

The TPS, SABATH, BAHD, and JA-biosynthesis-related protein sequences from *A. thaliana* (13-LOXs, AOSs, AOCs, OPRs, OPCLs, ACX1s, MFPs, KATs, and JMT) were obtained from the TAIR (https://www.arabidopsis.org/) database. For the TPS family, the hidden Markov model (HMM) profiles of the TPS domains (PF01397 and PF03936) were used as queries to search for predicted TPS proteins in the *J. sambac* genome using HMMER (version 3.0) [69] with E-value < 1E−10. The BLAST program in Tbtools [78] was also used to identify the jasmine TPSs, with all TPSs from *A. thaliana* used as the query. The candidate TPSs with E-value < 1E−5 were further searched against the PFAM (version 33.1) (https://pfam.xfam.org/) or NCBI CDD (https://www.ncbi.nlm.nih.gov/cdd/) databases to confirm the existence of TPS conserved domains in the candidates. The final TPS protein sequences of *J. sambac* were aligned using Clustal X (version 2.0), and the phylogenetic tree was constructed using IQ-TREE (version 1.6.9) by the maximum likelihood method with bootstrap values of 1000 replicates. The chromosomal distribution of *TPS* genes was illustrated with Tbtools according to the chromosome locations in the gff file. For the BAHD family, the HMM file of the BAHD domain (PF02458) was used as a query to search the candidate genes using HMMER (version 3.0) with E-value < 1E−10. SMART (https://smart.embl-heidelberg.de/) was used to further determine the domain PF02458 of BAHD, and multiple sequence alignments were performed to inspect the conserved HXXXD and DFGWG domains on the MAFFT website (https://mafft.cbrc.jp/alignment/server/). The final genes with the two domains (HXXXD and DFGWG) were identified as candidate *BAHD* genes. All heatmaps of gene expression were displayed using Tbtools [78]. For SABATH family and JA biosynthesis-related genes, the bidirectional BLAST was performed with a cutoff E-value < 1E−10, and then candidate genes were searched against Swiss-Prot and NCBI CDD databases for further confirmation. In addition, genes with tandem duplication were identified using MCScanX [76] and Tbtools [78].

### RNA-seq and transcriptomic analysis

The adult leaves were prodded with needles to simulate insect biting; 20 min after prodding, the WLs and NLs as well as FBs and FFs were collected and stored in liquid nitrogen, and then transferred to a freezer at −80 °C before RNA extraction. Three biological replicates (each treatment and each tissue) were conducted. RNA-seq libraries were constructed according to the manufacturer’s instructions and sequenced on the Illumina HiSeq 4000 platform at Novogene. After filtering the reads containing adapters, reads containing poly-N, and low-quality reads, the clean reads of each sample were mapped to the reference genome of *J. sambac* by HISAT2 [79] with the parameter “--dta”. Gene expression levels were calculated using reads per kilobase of transcript per million mapped reads (RPKM). Differential expression analysis among the samples was performed using DESeq2 (version 1.18.0) [80]. The DEGs were defined as those with adjusted *P* < 0.05 and |log_2_ fold change| > 1. GO enrichment analysis of DEGs was carried out using the *clusterProfiler* package (version 3.4.4) [81].

### Detection of metabolites in FBs and FFs by a widely-targeted metabolomics method

Sample preparation, extract analysis, and metabolite identification and quantification were conducted by Metware Biotechnology Co., Ltd. (Wuhan, China), according to their standard procedures. There is almost no fragrance released in the flowers at stage II (FBs, Figure 1A), whereas a large amount of fragrance is released at stage III (FFs, both semi-bloom and full-bloom, Figure 1A). Thus, we sampled the flowers at stage III to identify the released fragrance, and the flowers at stage II were used as the control. The FBs (12–15 buds per plant; stage II) and FFs (12–15 flowers per plant; stage III) were collected from three healthy jasmine plants (three biological replicates) at the flowering stage. These samples were immediately snap-frozen in liquid nitrogen and then stored in a freezer at −80 °C. The freeze-dried FBs and FFs were crushed using a mixer mill (Catalog No. MM 400, Retsch, Haan, Germany) with a zirconia bead for 1.5 min at 30 Hz. Powder (100 mg) was weighed and extracted overnight at 4 °C with 0.6 ml 70% aqueous methanol. After centrifugation at 10,000 *g* for 10 min, the extracts were absorbed (CNWBOND Carbon-GCB SPE Cartridge, 250 mg, 3 ml; ANPEL, Shanghai, China) and filtrated (SCAA-104, 0.22 μm pore size; ANPEL). Then, the sample extracts were analyzed using a ultra-performance LC–electrospray ionization–tandem MS (UPLC–ESI–MS/MS) system (UPLC: Shim-pack UFLC CBM30A, Shimadzu, Kyoto, Japan; MS: QTRAP 4500, ThermoFisher Scientific). For each sample, three biological replicates were independently analyzed. The analytical conditions were as follows: HPLC column, 1.8 µm, 2.1 mm × 100 mm (Waters ACQUITY UPLC HSS T3 C18); and solvent system, water (0.04% acetic acid):acetonitrile (0.04% acetic acid). The gradient program was as follows: 95:5 (v/v) at 0 min, 5:95 (v/v) at 11.0 min, 5:95 (v/v) at 12.0 min, 95:5 (v/v) at 12.1 min, 95:5 (v/v) at 14.0 min; flow rate, 0.35 ml/min; temperature, 40 °C; and injection volume: 4 μl. The effluent was connected to an ESI-triple Q–TRAP–MS.

MS analysis was performed according to a method described previously [82]. The ESI source operation parameters were as follows: ion source, turbo spray; source temperature, 550 °C; ion spray voltage, 5.5 Kv; ion source, gas I, gas II, and curtain gas set at 50, 60, and 30.0 psi, respectively; and high-pressure collision gas. Instrument tuning and mass calibration were performed with 10 and 100 μmol/l polypropylene glycol solutions in triple quadrupole (QQQ) and linear ion trap modes, respectively. The QQQ scans were acquired in multiple reaction monitoring (MRM) experiments with the collision gas (nitrogen) set to 5 psi.

Qualitative and quantitative analyses of metabolites were performed according to the methods of Li and colleagues [83]. Qualitative analyses of primary and secondary MS data were carried out according to the precursor ions (Q1), product ion (Q3) values, and retention time based on the self-built target standard database, Metware database (MWDB), by the widely targeted UPLC–ESI–MS/MS platform. Metabolite quantification was performed by MRM analysis via triple quadrupole MS.

### Detection of volatiles using gas chromatography**–**mass spectrometry

The FB and FF samples were used for gas chromatography–mass spectrometry (GC–MS) in the same manner as for LC–MS detection. The FBs and FFs stored at −80 °C were ground to powder in liquid nitrogen. The powder (1 g) was immediately transferred to a 20 ml headspace vial (Catalog No. 5183-4474, Agilent, Palo Alto, CA) containing 2 ml of saturated NaCl solution to inhibit any enzyme reaction. The vials were sealed using crimp-top caps with tetrafluoroethylene (TFE)-silicone headspace septa (Catalog No. 5183-4477, Agilent). At the time of solid-phase microextraction analysis, each vial was incubated at 60 °C for 10 min, then a 65-µm divinylbenzene/carboxen/polydimethylsiloxane fiber (Catalog No. 57310-U, Supelco, Bellefonte, PA) was exposed to the headspace of the sample for 20 min at 60 °C. After sampling, desorption of the VOCs from the fiber coating was carried out in the injection port of the GC apparatus (Model 7890B; Agilent) at 250 °C for 5 min in splitless mode. The identification and quantification of VOCs were carried out using a 7890B GC and a 7000D mass spectrometer (Agilent) equipped with a 5% phenyl-polymethylsiloxane capillary column (DB-5MS, 30 m × 0.25 mm × 1.0 μm; Agilent). Identification of volatile compounds was performed by comparison of the mass spectra with the data system library (NIST) and linear retention index.

### Detection of volatiles actively released from flowers on living plants

**Supplementary Figure S16:**
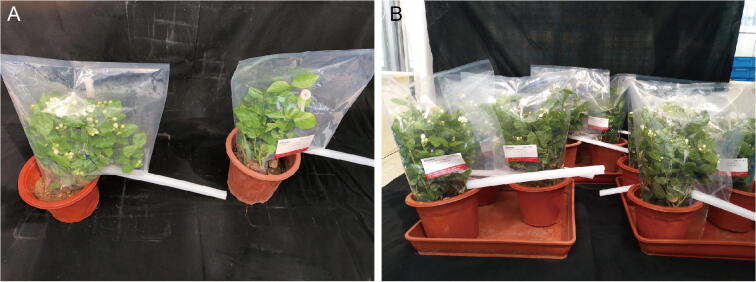
**Volatiles collection obtained with MonoTrap® DCC 18 A.** Single pot of *J. sambac* plant. Left: *J. sambac* plant with flowers. Right: Control plant; all flowers were removed. The arrow shows the MonoTrap® DCC 18 hanging on the branches next to the blooming flowers. **B**. Overview of *J. sambac* plants for the volatiles collection.

For GC–MS-based volatile detection on living plants, we selected six plants (three plants for FBs and another three for FFs) with similar numbers of flowers (about 12–15 FBs or FFs per plant) to collect the volatiles actively released from flowers on living plants. MonoTrap (DCC 18; GL Sciences, Tokyo, Japan) disks were used as absorbents for volatile collection. The aboveground parts of *J. sambac* plants were covered and fastened to a Teflon gas-sampling bag (5 l), with MonoTrap disks hanging on branches next to blooming flowers (Figure S16).

After 6 h of absorption, all MonoTrap disks were collected in sealed bottles (one disk per bottle). The disks were crushed under liquid nitrogen, and then carbon disulfide was used to elute and collect absorbed volatiles. The Exactive GC Orbitrap GC–MS system (ThermoFisher Scientific) coupled with a Tace1310 GC was used for metabolite analysis.

Extracts were liquid injected and separated by a DB-5 column using the following GC program: start at 40 °C, hold for 5 min, then increase the temperature to 280 °C at a rate of 5 °C/min. The scan range of 33–550 *m/z* was acquired with data-dependent MS/MS acquisition with 60,000 resolution under full scan mode. The source parameters were as follows: ion source temperature, 280 °C; MS transfer line, 250 °C.

MS/MS data were analyzed using TraceFinder analysis software (ThermoFisher Scientific). Data processing parameter settings were as follows: minimum peak width = 10 s, maximum peak width = 60 s, mzwid = 0.015, minfrac = 0.5, bw = 5, and signal/noise threshold = 5.

Chromatographic peaks were extracted from MS profiles, with mass tolerance of 10 ppm and a retention time window of 12 s.

Qualitative analysis of MS data profiles was performed using TraceFinder (version 4.1; ThermoFisher Scientific). An in-house database including standards and the NIST 2017 mass spectral library were both used for compound identification. Compounds were identified by comparison of the MS/MS fragment patterns, and the fragment ratio and retention time were then used in conjunction with the in-house database and NIST 2017 mass spectral library to further confirm the metabolites.

Quantitative analysis of MS data profiles was performed using Xcalibur (version 4.1; ThermoFisher Scientific). The chromatographic peaks of target compounds were extracted and identified using our in-house database, based on fragments, ratios, and retention time. The base peaks of spectra were then selected, and calibration curves of target compounds were used for quantitative detection and concentration calculation.

### Plant hormone extraction

Six plants in the full-bloom stage were selected for the measurement of JA-related compounds. For each plant, about 10–15 flowers (including FBs) and five leaves were collected. Wounding treatment was performed on leaves to simulate insect attacks. Three biological replicates for the treatment and tissue were used. The collected flower (FB and FF) and leaf (NL and WL) samples were immediately stored in liquid nitrogen. Then, the metabolites of the samples were extracted using a modified Wolfender method [84]. First, samples of 100 mg of flower and leaf powder obtained by crushing under liquid nitrogen were weighed and transferred to 2-ml centrifuge tubes with 10 µl of internal standards (10 µg/ml d5-JA). Second, 1.5 ml of extraction buffer (isopropanol:formic acid = 99.5:0.5, v/v) was added followed by vortexing to resuspend samples. After 15 min centrifugation at 14,000 *g*, the supernatants were dried in a vacuum centrifugal concentrator (CentriVap, Labconco, Kansas City, MO) and resuspended with 1 ml of methanol solvent (85:15, v/v). Then, a C18 SPE tube (100 mg, 1 ml; Sep-Pak C18 SPE Cartridge, Waters Technology, Shanghai, China) was used for sample purification, and a total of 1.5 ml of eluent was collected for each sample. Finally, the eluents were dried in a vacuum centrifugal concentrator (CentriVap, Labconco) and resuspended with 100 µl of methanol solvent (60:40, v/v).

### LC–MS for plant hormones

Positive/negative ionization mode data were acquired using an Acquity UPLC I-Class (Waters Technology) coupled to a 4500 QTRAP triple quadrupole mass spectrometer (AB Sciex, Concord, Canada) equipped with a 50 mm × 2.1 mm, 1.7 μm Acquity UPLC BEH C18 column (Waters Technology); 10 μl samples were loaded each time, and then eluted at a flow rate of 200 μl/min with initial conditions of 50% mobile phase A (0.1% formic acid in acetonitrile) and 50% mobile phase B (0.1% formic acid in water) followed by a 10 min linear gradient to 100% mobile phase A. The auto-sampler was set at 10 °C.

MS was operated separately in positive/negative ESI mode. The [M+H] or [M−H] of the analyte was selected as the precursor ion; precursor ion/product ion details for quantitation under MRM mode are shown in Table S20. The temperature of the ESI ion source was set to 500 °C. Curtain gas flow was set to 25 psi, collisionally activated dissociation gas was set to medium, and the ionspray voltage was (+) 5500 V for positive ionization mode and (−) 4500 for negative ionization mode with ion gases 1 and 2 set to 50 psi. Data acquisition and processing were performed using AB SCIEX Analyst (version 1.6.3; ThermoFisher Scientific).

### Vector construction, transformation, and measurement

The gene-specific primers of *JsTPS3* (JS6G20740), *JsAOC1* (JSCONTIG5G0140), and *JsAOS* (JS1G13140) were designed using Primer Premier 5.0 (Table S21). The coding regions of these three genes without a stop codon (TGA) were amplified from the cDNA fragments of *J. sambac* leaves. The plant expression vector pRI101-GFP was selected and cut at the *Bam*H I site. The *JsTPS3*, *JsAOC1*, and *JsAOS* fragments were inserted into pRI101-GFP to generate *35S::gene-GFP* via homologous recombination with Exnase II (Catalog No. C112-01, Vazyme Biotech, Nanjing, China). After sequencing validation, three recombinant plasmids, *35S::pRI101-JsTPS3-GFP*, *35S::pRI101-JsAOC1-GFP*, and *35S::pRI101-JsAOS-GFP*, were obtained. The empty vector was used as a control. To validate the functions of the three genes, the constructs and empty vector (control) were introduced into the *J. sambac* stem apex-induced calli using the *Agrobacterium*-mediated transient transformation method. About 4 days after transformation, the expression of the three genes in *J. sambac* calli (∼ 30 days of cultivation on growth medium) was checked and validated. Given the putative functions of *JsAOC1/JsAOS* in JA biosynthesis, the contents of JA-related compounds (OPDA, JA, JA-Ile, and MeJA) in the transgenic *J. sambac* calli were measured. The JA content measurement was the same as the LC–ESI–MS/MS measurement of flower and leaf samples. A total of five biological repeats were used for these measurements.

For measurement of ocimene contents, the powder (100 mg) of *JsTPS3*-transgenic and control calli was transferred immediately into a 20-ml headspace vial (Agilent). Then, 2 ml of saturated NaCl solution (containing 100 ng of 2-hepatone as an internal standard) was added to inhibit enzyme reactions. A 1.5-mm DVB/Carbon WR/PDMS SPME Arrow (CTC Analytics AG, Zwingen, Switzerland) was used for β-ocimene (*trans*-β-ocimene and β-ocimene) measurements. Each vial was heated at 60 °C for 10 min, and the SPME Arrow was then exposed to the headspace of the sample for 10 min at 60 °C. After sampling, desorption of the volatile metabolites from the Arrow coating was carried out in the injection port of the GC apparatus at 200 °C for 2 min in splitless mode. A Thermo Exactive GC Orbitrap equipped with Thermo Trace 1310 GC was used for identification and quantification, with a 30 m × 0.25 mm × 1.0 μm DB-5MS (5% phenyl-polymethylsiloxane) capillary column. The GC program was as follows: hold at 40 °C for 5 min, and then increase to 280 °C at a rate of 10 °C/min. The scan range of 33–550 *m/z* was acquired with data-dependent MS/MS acquisition, with a resolution of 60,000 in full scan mode. The source parameters were as follows: ion source temperature, 280 °C; and MS transfer line, 250 °C. The standard (Catalog No. S42430) was supplied by Shanghai Yuanye Bio-Technology (Shanghai, China). Data acquisition and processing were performed using TraceFinder analysis software (ThermoFisher Scientific).

### Protein expression and purification of JsTPS3

The full-length CDS of *JsTPS3* was amplified and inserted into the pGEX-6p-1 vector digested with *Bam*H I and *Eco*R I using Phanta Max Super-Fidelity DNA Polymerase (Catalog No. P505-d1, Vazyme Biotech) (Table S21). The construct *pGEX-6p-1-JsTPS3* was introduced into the *E. coli* strain Rosetta (DE3) (Vazyme Biotech). Then, 0.3 mM isopropyl-β-d-thiogalactopyranoside (IPTG) was added to induce protein expression for 12 h at 16 °C. The bacteria solution was centrifuged to collect cells at 10,000 r/min for 10 min, and the cells were then broken by ultrasonication. The GST-fused JsTPS3 recombinant protein was purified using a glutathione resin column (Catalog No. 786-310, GE Healthcare, Little Chalfont, UK) in accordance with the manufacturer’s instructions. The purified recombinant protein was further confirmed by SDS-PAGE. The protein concentration was measured using a BCA protein quantification kit (Catalog No. E112-02, Vazyme Biotech).

### Assays for enzyme activity of JsTPS3

Assays for JsTPS3 enzyme activity were performed according to methods described previously [85], [86]. Briefly, recombinant JsTPS3 proteins (10 μg and 15 μg) were incubated in 1 ml of assay buffer (30 mM HEPES pH 7.5, 5 mM DTT, 25 mM MgCl_2_) containing 60 μM GPP at 30 °C for 1 h, and then at 45 °C for 15 min. The purified GST (control) was also incubated under the same conditions. After incubation, the synthesized volatiles in each reaction were extracted with equal volume CH_2_Cl_2_ (containing 500 ng 2-hepatone as inner standard). β-ocimene content was determined by a Thermo Exactive GC Orbitrap equipped with Thermo Trace 1310 GC (30 m × 0.25 mm × 1.0 μm DB-5MS) in liquid injection mode. GC program was started at 40 °C for 5 min, and increased to 280 °C with a rate of 5 °C/min. All measurements were performed in triplicate.

### qRT-PCR analysis

qRT-PCR experiments were performed to examine the gene expression patterns in different organs, leaves after wounding, and transgenic calli according to procedures described previously [87]. NLs, FBs, and FFs were collected from at least three individuals for qRT-PCR. Leaves were sampled at 20 min, 1 h, 2 h, 4 h, 8 h, and 16 h after wounding. Transformed and control calli were also sampled. All samples were immediately snap-frozen in liquid nitrogen.

Each RNA sample was isolated using the Mini BEST plant RNA extraction kit (Catalog No. 9769, TaKaRa, Dalian, China) in accordance with the manufacturer’s protocol. The isolated RNA solution (10 μl) was reverse transcribed using the PrimeScript reverse transcriptase reagent kit with gDNA eraser (perfect real time) (Catalog No. RR047A, TaKaRa) in accordance with the manufacturer’s instructions. Gene-specific primers were designed using Primer (version 5.0) (Table S21). *Actin* was used as an internal reference. All reactions were performed in three biological replicates, and the comparative threshold cycle (Ct) was determined. Relative expression levels of the target genes were calculated with the 2^−ΔΔCt^ method.

### Statistical analysis

All statistical analyses were performed using SPSS software (version 23.0; IBM Corp., Armonk, NY) and R Studio (version 1.0.143; R Development Core Team, Vienna, Austria) with the Stats R package. The physiological data (floral fragrance measurements) and qRT-PCR data are presented as mean ± SD from at least three independent experiments (three biological replicates). In all analyses, *P* < 0.05 was taken to indicate statistical significance.

## Data availability

The genome assembly sequences and gene annotations have been deposited in the Genome Warehouse database [88] at the National Genomics Data Center (NGDC), Beijing Institute of Gemonics (BIG), Chinese Academy of Sciences (CAS) / China National Center for Bioinformation (CNCB) (GWH: GWHAZHY00000000), and are publicly accessible at https://ngdc.cncb.ac.cn/gwh. The raw datasets for genome assembly and transcriptome sequencing have been deposited in the Genome Sequence Archive [89] at the NGDC, BIG, CAS / CNCB (GSA: CRA008133, CRA005366, CRA005361, and CRA005359), and are publicly accessible at https://ngdc.cncb.ac.cn/gsa.

## Competing interests

The authors have declared no competing interests.

## CRediT authorship contribution statement

**Gang Chen:** Conceptualization, Methodology, Writing – original draft, Writing – review & editing. **Salma Mostafa:** Methodology, Data curation, Writing – original draft. **Zhaogeng Lu:** Conceptualization, Methodology, Data curation, Writing – original draft, Writing – review & editing. **Ran Du:** Methodology, Data curation, Writing – original draft. **Jiawen Cui:** Methodology. **Yun Wang:** Data curation. **Qinggang Liao:** Data curation. **Jinkai Lu:** Data curation. **Xinyu Mao:** Methodology. **Bang Chang:** Methodology. **Quan Gan:** Methodology. **Li Wang:** Supervision. **Zhichao Jia:** Data curation. **Xiulian Yang:** Data curation. **Yingfang Zhu:** Data curation. **Jianbin Yan:** Supervision, Project administration. **Biao Jin:** Conceptualization, Methodology, Supervision, Project administration, Funding acquisition. All authors have read and approved the final manuscript.

## Supplementary material

Supplementary Table S1 **Statistics of the *J. sambac* genome and gene annotation**

Supplementary Table S2 **Genome assembly using both PacBio reads and Hi-C data**

Supplementary Table S3 **Distribution of chromosomes in the assembled genome of *J. sambac***

Supplementary Table S4 **Quality assessment of the assembled genome of *J. sambac***

Supplementary Table S5 **Summary statistics of the annotated transposable elements (TEs) in the *J. sambac* genome**

Supplementary Table S6 **Summary statistic of annotated genes in the *J. sambac* genome. Genes were annotated by the combination of *de novo*, homology-based, and RNA-Seq data**

Supplementary Table S7 **Summary statistics of the functional genes of *J. sambac***

Supplementary Table S8 **Quality assessment of the completeness of gene annotation of *J. sambac* using BUSCOs**

Supplementary Table S9 **Annotated non-coding RNA in *J. sambac* genome**

Supplementary Table S10 **Functional annotation of the genes in *J. sambac***

Supplementary Table S11 **Expanded and contracted genes with annotations in *J. sambac***

Supplementary Table S12 **TPS genes containing at least one conserved domain in the genome of *J. sambac***

Supplementary Table S13 **Volatile metabolites (GC-MS) in FBs and FFs in *J. sambac***

Supplementary Table S14 **GC-MS based volatile metabolites released from FBs and FFs on living *J. sambac***

Supplementary Table S15 **Differential abundant terpene metabolites identified between the FBs and FFs in *J. sambac***

Supplementary Table S16 **Widely-targeted (UPLC-MS) metabolomics study of FBs and FFs in *J. sambac***

Supplementary Table S17 **Differentially abundant phenylpropanoid/benzenoid metabolites identified between the FBs and FFs in *J. sambac***

Supplementary Table S18 ***BAHD* genes containing two conserved domains in the genome of *J. sambac***

Supplementary Table S19 **JA biosynthetic genes identified in the genome of *J. sambac***

Supplementary Table S20 **Collision energies for different MRM pairs**

Supplementary Table S21 **Primers for homologous recombination and qRT-PCR of candidate genes in *J. sambac***
